# Leukotriene C_4_ is the major trigger of stress-induced oxidative DNA damage

**DOI:** 10.1038/ncomms10112

**Published:** 2015-12-11

**Authors:** Efrat Dvash, Michal Har-Tal, Sara Barak, Ofir Meir, Menachem Rubinstein

**Affiliations:** 1 Department of Molecular Genetics, The Weizmann Institute of Science, Rehovot 7610001, Israel

## Abstract

Endoplasmic reticulum (ER) stress and major chemotherapeutic agents damage DNA by generating reactive oxygen species (ROS). Here we show that ER stress and chemotherapy induce leukotriene C_4_ (LTC_4_) biosynthesis by transcriptionally upregulating and activating the enzyme microsomal glutathione-S-transferase 2 (MGST2) in cells of non-haematopoietic lineage. ER stress and chemotherapy also trigger nuclear translocation of the two LTC_4_ receptors. Acting in an intracrine manner, LTC_4_ then elicits nuclear translocation of NADPH oxidase 4 (NOX4), ROS accumulation and oxidative DNA damage. *Mgst*2 deficiency, RNAi and LTC_4_ receptor antagonists abolish ER stress- and chemotherapy-induced ROS and oxidative DNA damage *in vitro* and in mouse kidneys. Cell death and mouse morbidity are also significantly attenuated. Hence, MGST2-generated LTC_4_ is a major mediator of ER stress- and chemotherapy-triggered oxidative stress and oxidative DNA damage. LTC_4_ inhibitors, commonly used for asthma, could find broad clinical use in major human pathologies associated with ER stress-activated NOX4.

## Abstract (web summary)

Chemotherapeutic agents elicit ER and oxidative stress as part of their mode of action. Here the authors show that chemotherapy and ER stress trigger MGST2-based biosynthesis of LTC_4_, whose inhibition abolishes chemotherapy- and ER stress-triggered oxidative stress and DNA damage, resulting in the attenuation of cell death.

## Graphical abstract

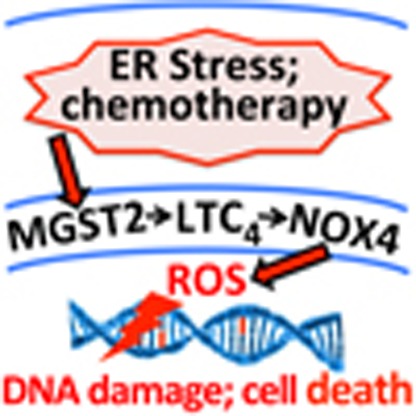

Endoplasmic reticulum (ER) stress, oxidative stress and oxidative DNA damage have been associated with major human pathologies, including neurodegenerative diseases, metabolic diseases, cardiovascular diseases and cancer^1^,^2^,^3^,^4^,^5^,^6^,^7^. Many physiological cues as well as chemotherapeutic agents trigger ER stress, initiating an evolutionarily conserved array of signalling pathways termed the unfolded protein response (UPR)^8^. Initial UPR is aimed at coping with the stress, whereas excessive stress triggers cell death. Among the several identified stress-triggered cell death mediators, C/EBPβ homologous protein (CHOP) is considered a major one^9^,^10^. CHOP activates several cell death mechanisms, for example, apoptosis mediated by inhibition of Bcl2, by activation of BAX and BAK and by induction of ER oxidase 1 (ERO1)^10^,^11^.

ER stress and oxidative stress are tightly associated events, triggering each other^12^. A major ER stress-triggered cell death mechanism involves CHOP-mediated accumulation of excess reactive oxygen species (ROS)^13^,^14^,^15^,^16^. Several mechanisms by which CHOP triggers oxidative stress were proposed. CHOP induces GADD34, a phosphatase that elevates messenger RNA (mRNA) translation of ER-destined proteins by dephosphorylation of p-eIF2α. This event combined with CHOP-induced upregulation of ERO1 elevates disulfide bond formation within the ER client proteins, leading to increased production of hydrogen peroxide as a byproduct^13^. However, ERO1-generated hydrogen peroxide does not trigger oxidative stress as it is rapidly cleared within the ER by glutathione peroxidase and does not permeate to other cellular compartments^17^. Transfer of calcium ions from the stressed ER to mitochondria could trigger apoptosis and subsequent release of abundant mitochondrial ROS to the cytoplasm^12^,^18^. Other studies implicated NADPH oxidase 2 (NOX2) in ER stress-triggered oxidative stress in macrophages and in the kidney^19^. Similarly, increased NOX4 activity was implicated in ER stress-triggered oxidative stress in smooth muscle cells^20^. However, the mechanism by which ER stress induces NOX4 is not known^18^,^21^.

Angiotensin II-induced leukotriene C_4_ (LTC_4_) was reported to trigger ROS accumulation^22^, prompting us to study whether LTC_4_ production is involved in ER stress-triggered oxidative stress. LTC_4_ has been extensively studied in the context of allergy and asthma^23^. Immunological cues trigger biosynthesis of LTC_4_ in mast cells by assembly of a biosynthetic complex at the nuclear envelope, consisting of cytosolic phospholipase A2 (cPLA2), 5-lipoxygenase (5-LO), 5-LO activating protein (FLAP) and LTC_4_ synthase (LTC_4_S). cPLA2 generates arachidonic acid by hydrolysis of membrane-associated phospholipids; 5-LO and FLAP oxidize arachidonic acid to form leukotriene A_4_, and LTC_4_S couples glutathione to leukotriene A_4_, thereby generating LTC_4_. The multidrug resistance protein 1 (MRP1) transporter then secretes cytosolic LTC_4_, and cell surface proteases further metabolize it by sequential cleavage of the γ-glutamyl and glycine residues off its glutathione segment, generating the more stable products leukotriene D_4_ (LTD_4_) and leukotriene E_4_ (LTE_4_). All three leukotrienes then bind at different affinities to two G-protein coupled receptors: CysLTR1 and CysLTR2, triggering pulmonary vasoconstriction and bronchoconstriction^24^.

Although LTC_4_S is expressed exclusively in cells of haematopoietic lineage such as mast cells, its isoenzyme, microsomal glutathione S-transferase 2 (MGST2), is ubiquitously expressed and functional in non-haematopoietic cells^25^,^26^,^27^. Unlike LTC_4_S, whose function has been extensively studied in the context of asthma and allergies, the physiological role of MGST2 has remained elusive^28^. Here, we reveal a previously unrecognized MGST2-LTC_4_ signalling cascade, activated by ER stress and by commonly used chemotherapeutic agents, which is the major inducer of oxidative stress, oxidative DNA damage and ROS-mediated cell death.

## Results

### ER stress triggers biosynthesis of LTC_4_

**Figure 1:**
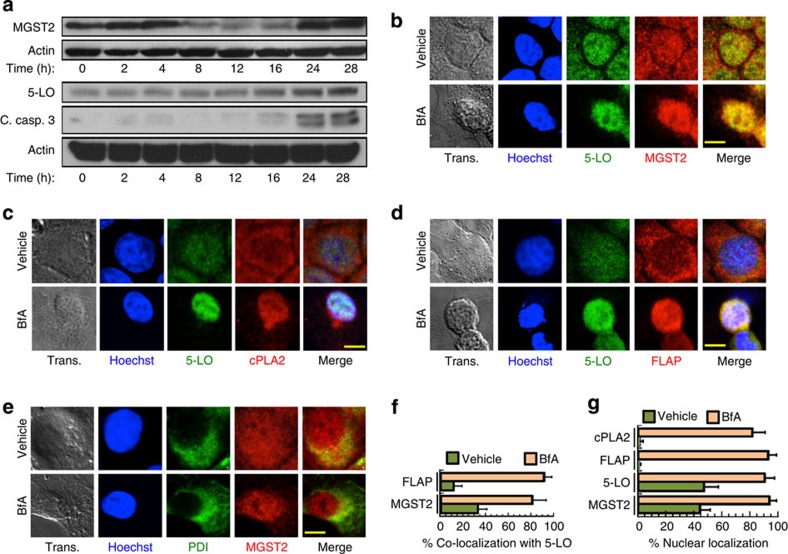
ER stress triggers expression and nuclear localization of proteins involved in LTC_4_ biosynthesis. (**a**) Immunoblot of proteins expressed in WISH cells at different times after induction of ER stress with BfA. Blots are representatives of three replicates. (**b**–**e**) Immunostain of the indicated proteins following treatment of WISH cells with vehicle or BfA. Trans. is transmission light microscopy. Nuclei were counterstained with Hoechst 33258 (Hoechst). Shown are merges of 5-LO and MGST2; 5-LO, Hoechst and cPLA2; Hoechst, 5-LO and FLAP; and MGST2 and the ER marker protein disulfide isomerase (PDI). Bars, 5 μm. (**f**) Quantification of per cent co-localization of FLAP and MGST2 with 5-LO, as determined by analysis of confocal microscopy images. *n*=6, *P*<0.0001 for both pairs. (**g**) Quantification of per cent nuclear localization of the indicated proteins as determined by analysis of confocal microscopy of the images shown in panels **b**–**e**. *n*≥6, *P*<0.0001 for all samples. Values in **f** and **g** represent the mean±s.d. Statistical significance was determined using one-way analysis of variance (ANOVA).

**Figure 2:**
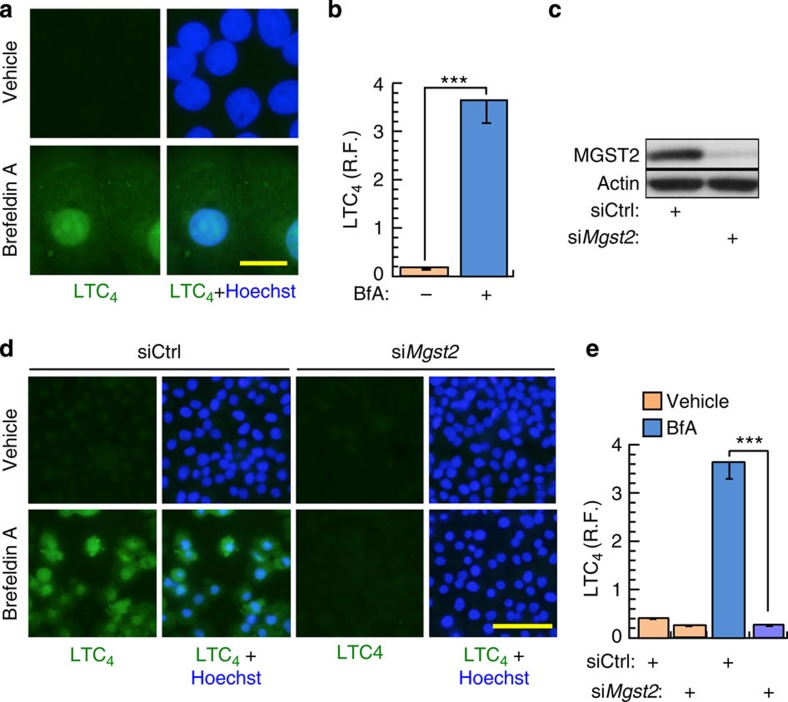
ER stress triggers MGST2-based biosynthesis of LTC_4_. (**a**,**b**) Immunostain of LTC_4_ after treatment of WISH cells with vehicle or BfA. R.F. is relative fluorescence intensity. Bar, 20 μm. *n*=3, ***P<0.001. (**c**) Immunoblot of MGST2 in WISH cell extracts following transfection with siControl or si*Mgst2*. (**d**,**e**) Immunostain of LTC_4_ after transfection of WISH cells with control siRNA or *Mgst2* siRNA, followed by vehicle or BfA. Bar, 100 μm. *n*=3, ***P<0.001. Values in **b** and **e** represent the mean±s.d. Statistical significance was determined using one-way ANOVA.

Upon triggering ER stress with Brefeldin A (BfA) or with tunicamycin (Tm) we found in several non-haematopoietic cell types that MGST2 and 5-LO, the rate-limiting enzyme of leukotriene biosynthesis, were downregulated during the early, protective phase of the UPR, and upregulated at the late, death-promoting phase of the UPR. Upregulation of MGST2 and 5-LO expression occurred concomitantly with elevation of cleaved caspase-3 and secretion to the culture media of the necrosis marker high mobility group protein 1 (HMGB1) (Fig. 1a, Supplementary Fig. 1a,b). ER stress triggered by BfA or by Tm also resulted in nuclear translocation and co-localization of MGST2, 5-LO, FLAP and cPLA2, thereby allowing assembly of an LTC_4_ biosynthetic machinery (Fig. 1b–f, Supplementary Fig. 1c–e). Untreated cells completely lacked nuclear FLAP and nuclear cPLA2, whereas ER stress led to near quantitative nuclear localization of these proteins (Fig. 1c,d,g). MGST2 and FLAP are transmembrane proteins, 5-LO is activated by binding to FLAP, and cPLA2 activation triggers its translocation and association with the nuclear envelope^29^. Therefore, assembly of these components into LTC_4_ biosynthetic machinery must have occurred at nuclear lipid bilayers such as the nuclear envelope and the nucleoplasmic reticulum, similarly to assembly of the analogous, LTC_4_S-based biosynthetic machinery of LTC_4_ in mast cells^24^. This ER stress-triggered assembly of MGST2-based biosynthetic machinery resulted in extensive production of LTC_4_ (Fig. 2a,b, Supplementary Fig. 1f). RNA interference (RNAi) experiments confirmed the key role of MGST2 in the ER stress-triggered biosynthesis of LTC_4_ (Fig. 2c–e). Inducible overexpression of MGST2 in HEK 293T cells was sufficient for triggering LTC_4_ production, thereby further demonstrating the role of MGST2 in LTC_4_ biosynthesis in cells of non-haematopoietic lineage under ER stress (Supplementary Fig. 1g–i).

### ER stress regulates LTC_4_ receptors expression and localization

**Figure 3:**
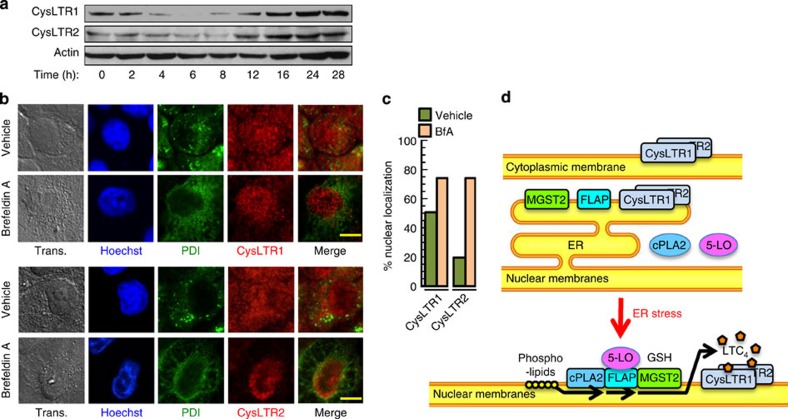
ER stress triggers expression and nuclear localization of the LTC_4_ receptors. (**a**) Immunoblot of the two LTC_4_ receptors, CysLTR1 and CysLTR2, expressed in WISH cells at different times after induction of ER stress with BfA. Blots are representatives of three replicates. (**b**) Immunostain of CysLTR1 and CysLTR2 after treatment with vehicle or BfA. Merge of PDI with CysLTR1 or with CysLTR2 is shown. Bar, 5 μm. (**c**) Quantification of per cent nuclear localization of the LTC_4_ receptors as determined by analysis of the confocal microscopy images shown in panel **b**. *n*≥6 for all samples, *P*<0.0001 for CysLTR2 and *P*<0.02 for CysLTR1. Values represent the mean±s.d. Statistical significance was determined using one-way ANOVA. (**d**) Scheme representing the translocation events triggered by ER stress, which initiate LTC_4_ biosynthesis and binding to its internalized receptors.

ER stress also regulated the expression of the two LTC_4_ receptors, CysLTR1 and CysLTR2, initially attenuating their level and upregulating it at the late, death-promoting phase of the UPR (Fig. 3a, Supplementary Fig. 2a). These receptors were initially localized both inside and outside the nucleus and following ER stress they were localized mainly at the nucleus (Fig. 3b,c, Supplementary Fig. 2b), suggesting that LTC_4_ action following ER stress is mostly intracrine. Figure 3d schematically represents this previously unrecognized ER stress-triggered MGST2-LTC_4_ pathway.

### ER stress-induced ROS production is mediated by LTC_4_

**Figure 4:**
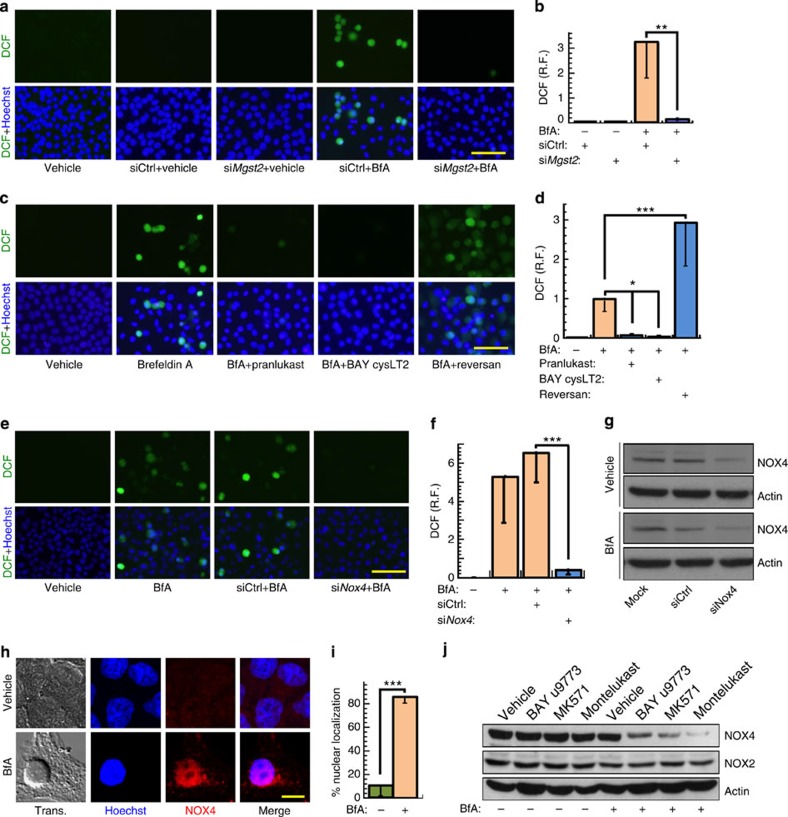
ER stress-generated LTC_4_ triggers NOX4-mediated ROS accumulation in WISH cells. (**a**,**b**) ROS detection using dichloro-dihydrofluorescein diacetate (DCFH-DA, hydrolysed and oxidized by ROS to dichlorofluorescein (DCF)) in cells transfected with control siRNA or *Mgst2* siRNA, and then treated with vehicle or BfA. Bar, 100 μm. *n*=4, **P<0.01. (**c**,**d**) ROS detection in cells treated with vehicle or BfA in the absence or presence of LTC_4_ receptor antagonists or the MRP1 transporter inhibitor reversan. Treatment with reversan or the receptor antagonists alone did not induce ROS. Bar, 100 μm. *n*=4, *P<0.02; ***P<0.001. (**e**,**f**) ROS detection and quantification in cells transfected with control siRNA or *Nox4* siRNA, and then treated with vehicle or BfA. Bar, 100 μm. *n*=5, ***P<0.001. (**g**) Immunoblot of NOX4 in extracts of cells transfected with control siRNA or *Nox4* siRNA, and then treated with vehicle or BfA. (**h**) Immunostain of NOX4 in cells treated with vehicle or BfA. Merge of Hoechst and NOX4 is shown. Bar, 5 μm. (**i**) Per cent nuclear localization of NOX4 as determined by analysis of confocal images shown in **e**. *n*=8, ***P<0.0001. (**j**) Immunoblot of NOX4 and NOX2 in extracts of cells treated with the indicated LTC_4_ receptor antagonists together with vehicle (−) or BfA (+). Blots **g** and **j** are representatives of three replicates. Values in **b**,**d**,**f** and **i** represent the mean±s.d. Statistical significance was determined using one-way ANOVA.

We employed several independent approaches to study the possible role of MGST2 and LTC_4_ in ER stress-triggered oxidative stress. Effective knockdown of *Mgst2* mRNA (Fig. 2c) abolished ER stress-triggered ROS accumulation (Fig. 4a,b, Supplementary Fig. 3a,b). To test if ROS accumulation was due to glutathione depletion by MGST2 or by downstream action of its product, LTC_4_, we employed LTC_4_ inhibitors. Because LTC_4_ and its metabolites inflict the symptoms of asthma, several inhibitors of leukotriene biosynthesis, transport or activity are available. The CysLTR1 antagonists pranlukast and montelukast, the CysLTR2 antagonist BAY cysLT2 and the dual receptor antagonist BAY u9773 independently abrogated ER stress-triggered ROS accumulation (Fig. 4c,d, Supplementary Fig. 3c–f), demonstrating that MGST2-generated LTC_4_ is the major upstream mediator of ER stress-triggered oxidative stress. The MRP1 transporter inhibitor reversan augmented ROS accumulation (Fig. 4c,d), indicating that LTC_4_ activity is mostly intracrine, in contrast with LTC_4_S-generated LTC_4_ in mast cells, which mainly acts on external target cells. This result was consistent with the observed nuclear translocation of the two LTC_4_ receptors under stress. Overexpression of MGST2 was sufficient for inducing ROS accumulation, thereby further validating its significant role in triggering oxidative stress (Supplementary Fig. 3g,h).

We then studied which ROS-generating mechanism is activated by the MGST2-LTC_4_ pathway. NADH/NADPH oxidases (NOXs) are major cellular ROS-producing enzymes^30^,^31^. Of these, ER stress was found to trigger oxidative stress by upregulating NOX2 and NOX4 activity^19^,^20^. Indeed, RNAi of *Nox4* significantly attenuated ER stress triggered ROS accumulation (Fig. 4e–g, Supplementary Fig. 4a). We then found that ER stress triggered nuclear translocation of NOX4 (Fig. 4h,i). In line with its ability to induce LTC_4_ and ROS even without ER stress, inducible overexpression of MGST2 also triggered nuclear translocation of NOX4 (Supplementary Fig. 4b). Moreover, treatment of cells with LTC_4_ receptor antagonists reduced NOX4 levels but not NOX2 levels under ER stress (Fig. 4j, Supplementary Fig. 4c). Similarly, *Mgst2* knockdown greatly inhibited NOX4 expression but not NOX2 upon ER stress (Supplementary Fig. 4d). Furthermore, *Mgst2* knockdown significantly inhibited nuclear translocation of NOX4 under ER stress (Supplementary Fig. 4e,f). Taken together, these results suggest that the MGST2-LTC_4_ pathway activates NOX4 or at least prevents its degradation under ER stress.

### LTC_4_ mediates ER stress-triggered oxidative DNA damage

**Figure 5:**
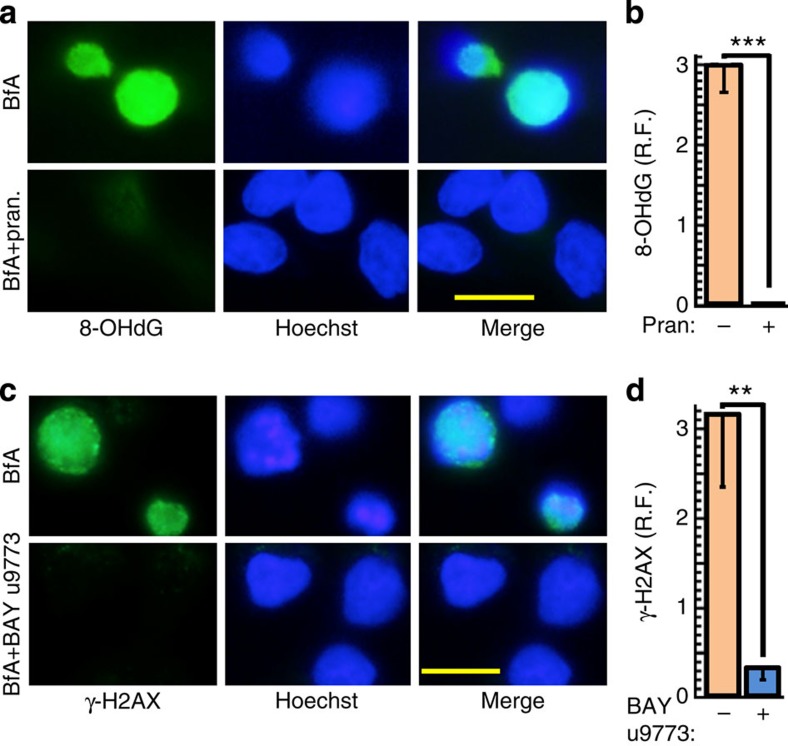
The MGST2-LTC_4_ pathway triggers oxidative DNA damage. (**a**,**b**) Immunostain of the oxidative DNA damage marker 8-OHdG in cells treated with vehicle or BfA in the absence or presence of pranlukast (pran.). Bar, 20 μm. *n*=3, ***P<0.0001. (**c**,**d**) Immunostain of the dsDNA break marker γ-H2AX in cells treated with vehicle or BfA in the absence or presence of BAY u9773. Bar, 20 μm. *n*=3, **P<0.005. Values in **b** and **d** represent the mean±s.d. Statistical significance was determined using one-way ANOVA.

NOX4 is implicated in oxidative DNA damage^32^,^33^, suggesting that the MGST2-LTC_4_ pathway might also be involved in triggering oxidative DNA damage. Indeed, LTC_4_ receptor antagonists and *Mgst2* knockdown abolished ER stress-triggered oxidative DNA damage, as determined by immunostaining of 8-hydroxy-2′-deoxy guanosine (8-OHdG) residues within the nuclear DNA, as well as γ-H2AX, the marker of nuclear dsDNA breaks (Fig. 5, Supplementary Fig. 4e–h). These findings elucidate the critical role of ER stress-generated LTC_4_ in triggering NOX4-mediated oxidative DNA damage following ER stress.

### MGST2 activation is downstream of CHOP

CHOP is induced by ER stress before other mediators of cell death. Indeed CHOP expression was apparent in WISH cells already at 8–12 h (Supplementary Fig. 5a), whereas MGST2 re-induction was apparent only at 24 h (Fig. 1a). We found that RNAi of *Chop* mRNA significantly attenuated *Mgst2* mRNA expression under ER stress, placing *Mgst2* transcriptional induction at the late, death-promoting phase of the UPR and downstream of CHOP (Supplementary Fig. 5b). Similarly, RNAi of *Chop* mRNA greatly attenuated MGST2 and NOX4 expression at the protein level, whereas NOX2 expression was not regulated by CHOP (Supplementary Fig. 5c,d). Furthermore, compared with wild-type (WT) mouse hepatocytes, *Chop* deficient hepatocytes^34^ exhibited reduced nuclear translocation of MGST2 under ER stress (Supplementary Fig. 5e). We then found that inhibition of LTC_4_ biosynthesis by zileuton, as well as inhibition of LTC_4_ binding to its receptors, greatly attenuated ER stress-triggered CHOP induction, whereas overexpression of MGST2 upregulated CHOP expression under ER stress (Supplementary Fig. 5f,g). These observations elucidate a positive feedback loop between CHOP and MGST2 in cells of non-haematopoietic lineage. A similar positive feedback loop between CHOP and NOX2 was previously reported in macrophages following ER stress^19^.

### LTC_4_ mediates ER stress-triggered cell death

**Figure 6:**
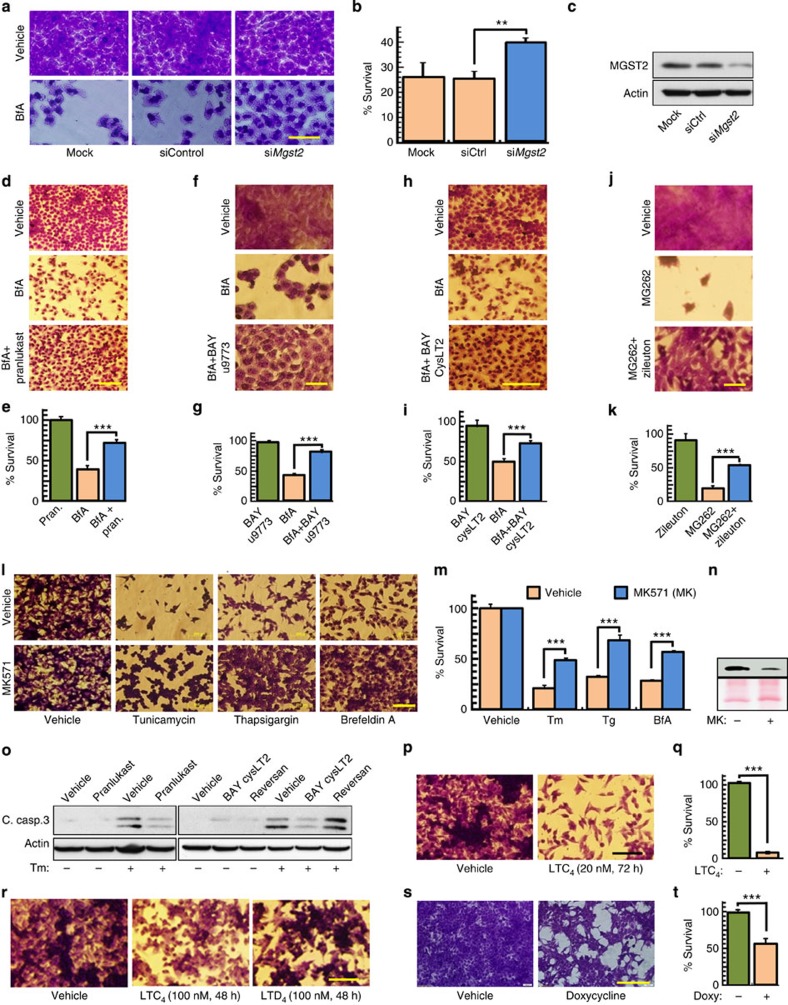
The MGST2-LTC_4_ pathway elicits ER stress-triggered cell death. Survival was determined by crystal violet staining and is relative to vehicle-treated cells. (**a**,**b**) Survival of WISH cells transfected with the indicated siRNA, treated with BfA (0.5 μg ml^−1^, 24 h). Bar, 100 μm. *n*=3, **P<0.02. (**c**) Immunoblot of MGST2 in extracts of WISH cells treated as in **a**. (**d**,**e**) Survival of WISH cells treated with BfA and pranlukast. Bar, 200 μm. *n*=3, ***P<0.001. (**f**,**g**) Survival of human HaCaT pre-keratinocytes treated with BfA (1.3 μg ml^−1^, 48 h) and BAY u9773 (80 nM). Bar, 100 μm. *n*=4, ***P<0.0001. (**h**,**i**) Survival of WISH cells treated with BfA (48 h) and BAY cysLT2. Bar, 500 μm. *n*=4, ***P<0.001. (**j**,**k**) Survival of HaCaT pre-keratinocytes treated with the proteasome inhibitor MG262 (0.05 μM) and zileuton. Bar, 50 μm. *n*=4, ***P<0.001. (**l**,**m**) Survival of B16 cells treated with Tm, thapsigargin (Tg) or BfA (1.3 μg ml^−1^) and the CysLTR1 antagonist MK571. Bar, 200 μm. *n*=4, ***P<0.001. (**n**) Immunoblot of the necrosis marker HMGB1 (top panel) in media of B16 cells treated with BfA (1.3 μg ml^−1^) and MK571 (MK). Ponceau S staining served as loading control. (**o**) Immunoblot of cleaved caspase-3 in extracts of WISH cells treated with Tm (2 μg ml^−1^, 48 h) and the indicated inhibitors. (**p**,**q**) Survival of B16 cells treated with LTC_4_. Bar, 200 μm. *n*=3, ***P<0.001. (**r**) Survival of B16 cells treated with LTC_4_ or LTD_4_. Bar, 200 μm. (**s**,**t**) Survival of HEK 293T cells stably transfected with Tet-inducible *Mgst2* expression vector, treated with doxycycline (2 μg ml^−1^, 48 h). Bar, 200 μm. *n*=3, ***P<0.001. Immunoblots **c**,**n** and **o** are representatives of three replicates. Values in **b**,**e**,**g**,**i**,**k**,**m**,**q** and **t** represent the mean±s.d. Statistical significance was determined using one-way ANOVA.

Our finding that MGST2 is downstream of CHOP prompted us to study the role of MGST2, LTC_4_ and NOX4 in ER stress-triggered cell death. Knockdown of *Mgst2*, as well as LTC_4_ receptor antagonists, significantly attenuated ER stress-triggered cell death, in correlation with lower release of HMGB1 and reduced cleavage of caspase-3 (Fig. 6a–o). Unlike the effect of LTC_4_ receptor antagonists, inhibition of the LTC_4_ transporter MRP1 by reversan did not reduce cleaved caspase-3 levels, thereby further demonstrating the intracrine nature of LTC_4_ action under stress (Fig. 6o). In line with these results, exogenously added LTC_4_ was cytotoxic, but at a much slower kinetics, and the outer cell membrane-generated LTD_4_ was even less toxic than LTC_4_ (Fig. 6p–r). As observed with nuclear translocation of NOX4 and ROS production, inducible overexpression of MGST2 triggered cell death even without ER stress (Fig. 6s,t). NOX4 was implicated in ER stress-triggered apoptosis^20^. Indeed, RNAi study revealed that NOX4-mediated oxidative stress plays a significant role in ER stress-triggered necrosis and apoptosis (Supplementary Fig. 6). These experiments indicate that the late ER stress-activated MGST2-LTC_4_ pathway is one of the major routes leading to cell death, mediated by intracrine action of LTC_4_ and involving oxidative stress, inflicted by LTC_4_-triggered activation of NOX4.

### *Mgst2* deficiency reduces oxidative DNA damage and cell death

**Figure 7:**
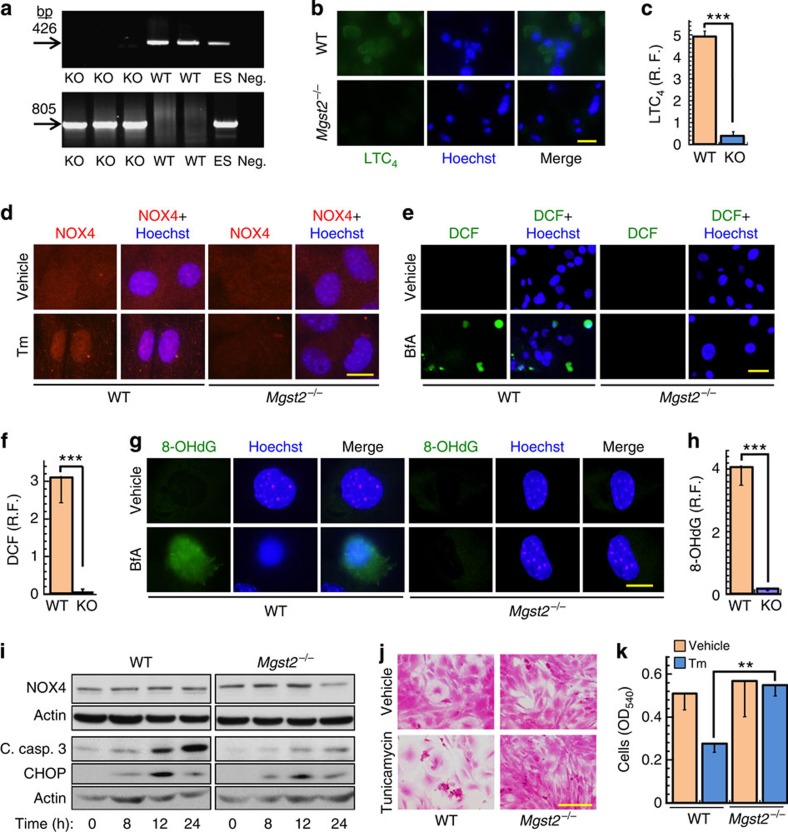
*Mgst2* deficiency attenuates ER stress-triggered oxidative stress, DNA damage and apoptosis. (**a**) Agarose gel electrophoresis of PCR products obtained by amplification of DNA isolated from tail ends of WT and homozygous *Mgst2*-deficient (KO) mice. DNA of heterozygous ES cells (ES) and negative PCR control (Neg.) are also shown. The 426-bp band corresponds to WT DNA sequence from the *Mgst2* gene. The 805-bp band corresponds to the mutated allele. (**b**,**c**) Immunostain of LTC_4_ in WT and *Mgst2*-deficient MEFs at passage 2 following treatment with BfA. Bar, 20 μm. *n*=3, ***P<0.0001. (**d**) Immunostain of NOX4 in WT and *Mgst2*-deficient MEFs at passage 2, treated with vehicle or Tm (4 μg ml^−1^, 24 h). Bar, 20 μm. This image is a representative of five replicates. (**e**,**f**) ROS detection using DCFH-DA in primary WT and *Mgst2*-deficient (KO) MEFs, treated with vehicle or BfA (0.25 μg ml^−1^). Bar, 20 μm. *n*=4, ***P<0.001. (**g**,**h**) Immunostain of 8-OHdG in primary WT and *Mgst2*-deficient MEFs, treated with vehicle or BfA. Bar, 20 μm. *n*=3, ***P<0.001. (**i**) Immunoblot of the indicated proteins in WT and *Mgst2*-deficient primary MEFs, treated with Tm (3 μg ml^−1^). (**j**) Cultures of WT and *Mgst2*-deficient primary MEFs, treated with vehicle or Tm (2 μg ml^−1^) and then stained with crystal violet. Bar, 50 μm. (**k**) Relative viability as determined by neutral red staining of MEFs, treated as in **j**. *n*=3, **P=0.005. Images **a** and **i** are representatives of at least three replicates. Values in **c**,**f**,**h** and **k** represent the mean±s.d. Statistical significance was determined using one-way ANOVA.

We then established homozygous *Mgst2* deficient mice and obtained murine embryonic fibroblasts (MEFs) of these mice (Fig. 7a). In contrast with its effect in primary WT MEFs, ER stress did not trigger the biosynthesis of LTC_4_ in the *Mgst2* deficient MEFs, thereby, further demonstrating the essential role of MGST2 in LTC_4_ biosynthesis under stress (Fig. 7b,c). Compared with WT MEFs, NOX4 expression at late ER stress was only slightly lower in *Mgst2*-deficient primary MEFs (Fig. 7d,i). However, ER stress triggered nuclear translocation of NOX4 in WT primary MEFs, and not in *Mgst2*-deficient primary MEFs (Fig. 7d). ER stress triggered ROS accumulation and subsequent DNA damage in WT MEFs but not in *Mgst2*-deficient MEFs (Fig. 7e–h). Furthermore, apoptosis and cell death were significantly reduced in the *Mgst2*-deficient MEFs despite induction of CHOP (Fig. 7i–k). Exogenous LTC_4_ added at time=0 restored ER stress-triggered cell death of *Mgst2*-deficient MEFs, bringing it on par with that of WT MEFs (Supplementary Fig. 7). This complete restoration of BfA toxicity was probably possible due to the longer exposure time to exogenous LTC_4_ as compared with that of the stress-induced LTC_4_.

### *Mgst2* deficiency & LTC_4_ antagonist attenuate mouse morbidity

**Figure 8:**
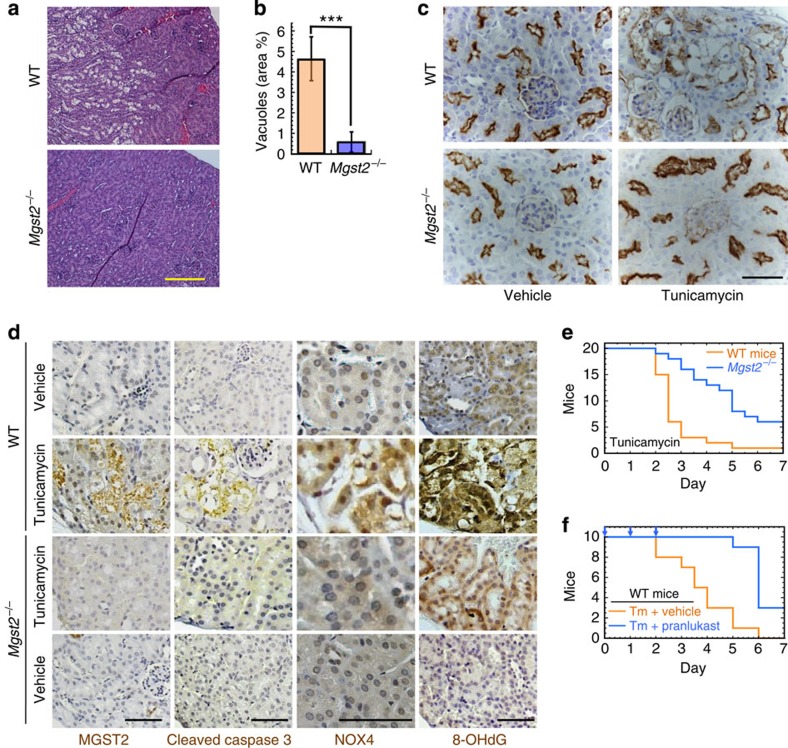
*Mgst2* deficiency and LTC_4_ inhibition attenuate ER stress-triggered oxidative damage to mouse kidneys and mouse morbidity. (**a**) Haematoxylin–eosin stained kidney slices from WT and *Mgst2*-deficient mice given a single dose of Tm (1.5 mg kg^−1^, ip) at time=0. Kidneys were removed and processed on day 4. The image is a representative of 10 kidneys obtained from 5 mice per group. Bar, 200 μm. (**b**) Quantification of per cent areas of vacuoles representing damage to kidney proximal tubules shown in **a**. *n*=5, ***P<0.001. Values represent the mean±s.d. Statistical significance was determined using one-way ANOVA. No vacuoles were observed in kidneys of untreated mice. (**c**) Immunohistochemical stains of proximal tubules (brown) using anti-aminopeptidase A in kidney sections from WT and *Mgst2*-deficient mice treated with Tm as in **a**. Nuclei were counterstained with haematoxylin (grey-blue). Bar, 50 μm. (**d**) Immunohistochemical stains of the indicated markers in kidney sections as in **c**. Figures are representatives of kidneys from three mice. Bars, 50 μm. (**e**) Survival of WT and *Mgst2*-deficient 129/Sv mice (20 per group) given Tm (2.5 mg kg^−1^, ip) at time=0. *P*=0.0393. (**f**) Survival of WT 129/Sv mice (10 per group) given Tm (1.5 mg kg^−1^, ip) at time=0 and daily administrations of either vehicle or pranlukast (ip, 1 mg kg^−1^, vertical arrows). *P*=0.0009. The statistical significance was determined in **e** and **f** using the Gehan–Breslow–Wilcoxon test.

To study the role of MGST2 in ER stress *in vivo*, we employed the mouse model of Tm-triggered acute kidney injury^35^. Tm administration gave rise to necrotic vacuoles mainly in the juxtamedular region of the kidney cortex in WT mice (Fig. 8a). Compared with WT mice, the area of the necrotic vacuoles was significantly reduced in the kidneys of *Mgst2*-deficient mice (Fig. 8a,b). Immunohistochemical staining using anti aminopeptidase A, a specific marker of renal proximal tubular cells^36^, revealed extensive destruction of these cells in the Tm-treated WT kidneys but not in the *Mgst2*-deficient kidneys (Fig. 8c). Cleaved caspase-3 and nuclear NOX4 were present in kidney sections of Tm-treated WT mice but not in the kidneys of Tm-treated *Mgst2*-deficient mice, leading to greatly reduced oxidative DNA damage (Fig. 8d). NOX4 was strongly induced in ER-stressed WT-mouse kidneys but not in kidneys of the *Mgst2*-deficient mice. The induction of NOX4 by ER stress in the kidneys but not in other cell types was probably due to the high basal expression level of NOX4 in the kidney compared with that in other organs and tissues^37^.

We then found that *Mgst2* deficient mice were significantly more resistant than WT mice towards Tm-triggered morbidity and mortality (Fig. 8e). Furthermore, inhibition of LTC_4_ with the CysLTR1 antagonist pranlukast significantly reduced the morbidity and mortality of Tm-treated WT mice (Fig. 8f). Taken together, these studies confirm the role of the MGST2-LTC_4_ pathway in mediating ER stress-triggered kidney damage, apoptosis, oxidative DNA damage and ER stress-triggered morbidity *in vivo*.

### Chemotherapeutic agents and the MGST2-LTC_4_ pathway

**Figure 9:**
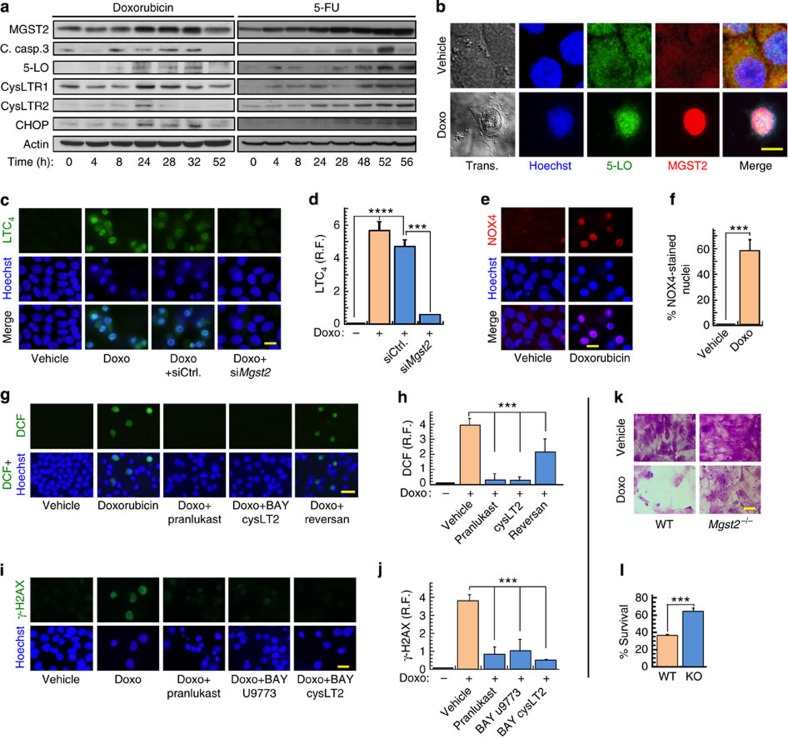
Chemotherapy-activated MGST2-LTC_4_ pathway triggers NOX4-mediated oxidative DNA damage and cell death. (**a**) Immunoblot of the indicated proteins in extracts of WISH cells treated with doxorubicin (5 μM) or 5-FU (20 μg ml^−1^) for the indicated times. (**b**) Immunostain of 5-LO and MGST2 in WISH cells treated with vehicle or doxorubicin (Doxo, 5 μM). Nuclei were counterstained with Hoechst. Trans. is transmission light microscopy. All image channels except the transmission light microscopy were merged. Bar, 5 μm. (**c**,**d**) Immunostain of LTC_4_ in WISH cells pre-treated with vehicle, siControl or si*Mgst2*, followed by vehicle or doxorubicin (1 μM, 36 h). Nuclei were counterstained with Hoechst. Bar, 20 μm. *n*=3, ****P<0.0001, ***P<0.001. (**e**,**f**) Immunostain of NOX4 in cells treated with vehicle or doxorubicin. Bar, 20 μm. *n*=3, ***P<0.001. (**g**,**h**) ROS detection with DCFH-DA in cells treated with vehicle or doxorubicin (2.5 μM, 48 h) in the absence or presence of the indicated inhibitors. Bar, 50 μm. *n*=6, ***P<0.0001. (**i**,**j**) Immunostain of the dsDNA break marker γ-H2AX in cells treated with doxorubicin (5 μM, 48 h) in the absence or presence of the indicated LTC_4_ receptor antagonists. Bar, 20 μm. *n*=3–4, ***P<0.0001. (**k**,**l**) Survival of primary WT and *Mgst2*-deficient MEFs, treated with vehicle or doxorubicin (10 μM) and then stained with crystal violet. Bar, 50 μm. Viabilities of vehicle-treated WT and and KO MEFs were taken as 100% survival, respectively. *n*=3, ***P<0.005. Images **a** and **b** are representatives of three replicates. Values in **d**,**f**,**h**,**j** and **l** represent the mean±s.d. Statistical significance was determined using one-way ANOVA.

Doxorubicin is an effective antineoplastic agent, killing rapidly dividing tumour cells by several mechanisms, including inhibition of topoisomerase 2 (ref. 38). However, the use of doxorubicin is limited by its severe cardiotoxicity, attributed to oxidative stress^39^. Doxorubicin triggers ROS accumulation in cardiac myocytes mainly by NOXs (ref. 40). Other chemotherapeutic agents, such as 5-fluorouracil (5-FU), vincristine and bortezomib generate ROS as well^41^,^42^,^43^, suggesting that chemotherapy-triggered ER stress^44^ might be a common underlying mechanism by which chemotherapy triggers oxidative stress. Indeed, doxorubicin and 5-FU induced the expression of MGST2, 5-LO, the two LTC_4_ receptors, cleaved caspase-3 and CHOP (Fig. 9a). Similarly to the specific ER stress inducers, doxorubicin, 5-FU and bortezomib elicited translocation of the LTC_4_ biosynthetic machinery and its receptors to the nucleus (Fig. 9b, Supplementary Fig. 8). Doxorubicin induced MGST2-mediated biosynthesis of LTC_4_ and translocated NOX4 to the nucleus (Fig. 9c–f).

Remarkably, LTC_4_ receptor antagonists abolished doxorubicin-triggered ROS accumulation and significantly inhibited nuclear DNA damage, as determined by staining with DCFH-DA and immunostaining of γ-H2AX (Fig. 9g–j). In addition, *Mgst2* deficiency, LTC_4_ receptor antagonists and 5-LO inhibition attenuated the cytotoxicity of all four chemotherapeutic agents (Fig. 9k,l, Supplementary Fig. 9a–i). As with BfA, exogenous LTC_4_ added at time=0 restored doxorubicin-triggered death of *Mgst2*-deficient MEFs, bringing it on par with that of WT MEFs (Supplementary Fig. 9j,k). These results demonstrate that a broad range of chemotherapeutic agents, acting by different mechanisms, activate the MGST2-LTC_4_ pathway. Importantly, this pathway is the major mediator of chemotherapy-triggered oxidative stress, oxidative DNA damage and oxidative stress-triggered cell death.

### Chemotherapy activates the MGST2-LTC_4_ pathway *in vivo*

**Figure 10:**
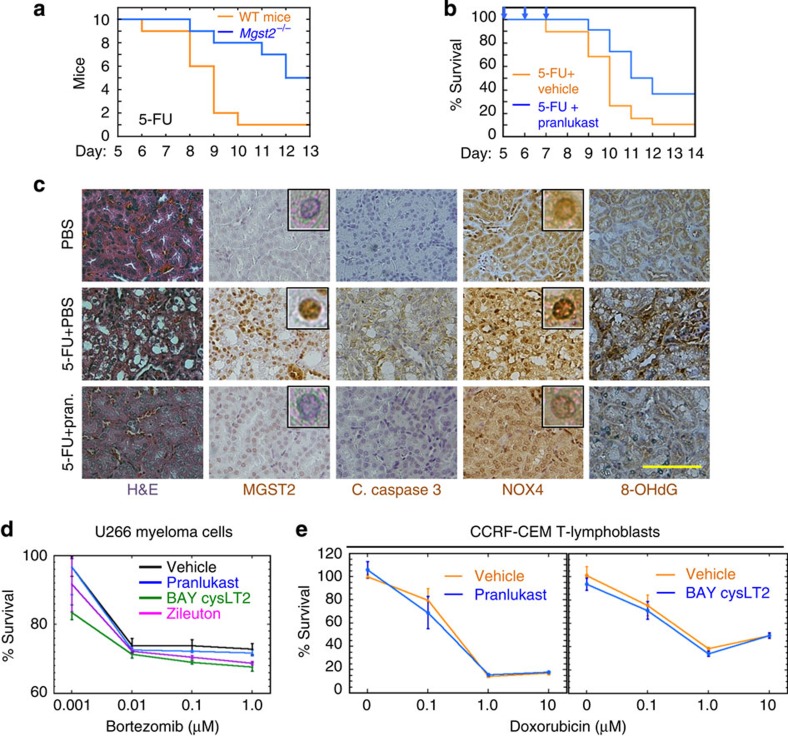
*Mgst2* deficiency and LTC_4_ inhibition attenuate 5-FU-triggered DNA damage and toxicity. (**a**) Survival of WT and *Mgst2*-deficient mice (10 per group) given 5-FU (300 mg kg^−1^, ip) at time=0. *P*=0.0085. (**b**) Survival of WT 129/Sv mice (18 per group) treated with 5-FU as in **a**. Vehicle or pranlukast (1 mg kg^−1^) were administered at days 0, 1, 2, 5, 6 and 7. *P*=0.032. The statistical significance was determined in **a** and **b** using the Gehan–Breslow–Wilcoxon test. (**c**) Haematoxylin–eosin (H&E) stain and immunostain of the indicated proteins and 8-OHdG in kidney slices of WT mice treated with 5-FU (300 mg kg^−1^, ip at time=0) followed by five administrations of PBS or pranlukast (Pran., 3 mg kg^−1^, ip) as in **b**. Kidneys were processed at day 13. Bar, 50 μm. Insets: enlarged images showing immunostained nuclei. The images of kidney slices are representatives of slices from three mice per group. (**d**) Survival of U266 myeloma cells treated with bortezomib alone or together with the indicated LTC_4_ inhibitors (10 μM each). *n*=3. (**e**) Survival of CCRF-CEM T-cell leukaemia lymphoblasts treated with doxorubicin together with the indicated LTC_4_ inhibitors (10 μM each). *n*=3. No significant differences were found between vehicle and any of the inhibitors in **d** and **e**. Statistical significance was determined using one-way ANOVA.

We then studied the impact of *Mgst2* deficiency and LTC_4_ inhibition on the toxicity of 5-FU in mice. *Mgst2* deficient mice were significantly more resistant than WT mice to 5-FU, and pranlukast significantly attenuated 5-FU-triggered morbidity in WT mice (Fig. 10a,b). Immunohistochemical examination revealed that pranlukast inhibited 5-FU-triggered formation of necrotic vacuoles, induction of MGST2, activation of caspase 3, nuclear translocation of NOX4 and oxidative DNA damage in kidneys of 5-FU-treated WT mice (Fig. 10c). MGST2 is not expressed in cells of haematopoietic lineage^25^, implying that the MGST2-LTC_4_ pathway will not be activated in these cells. Indeed, LTC_4_ inhibitors did not compromise the cytotoxicity of bortezomib in human myeloma cells, or that of doxorubicin in T-leukaemia cells (Fig. 10d,e). These results suggest that the MGST2-LTC_4_ pathway is not activated by chemotherapy in cells of haematopoietic lineage.

## Discussion

Many chemotherapeutic agents trigger both extensive ER stress and oxidative stress as part of their mechanism of action^45^,^46^. The present study describes a previously unrecognized ER stress-activated MGST2-LTC_4_ pathway as the major executor of chemotherapy-triggered ROS generation and subsequent DNA damage. The MGST2-LTC_4_ pathway appears to be quite general, as we have identified it in numerous human and mouse cell types, including epithelial cells, fibroblasts and pre-keratinocytes. We have further demonstrated its role in ER stress and chemotherapy-triggered oxidative cell death *in vivo* using both genetic and pharmacological mouse models.

The components of this pathway include MGST2-based biosynthetic machinery of LTC_4_ and the two LTC_4_ receptors. Our findings that expression of genes encoding these components is attenuated during the early, pro-survival phase of the UPR, and is restored and further induced at the late phase, links these genes with the death-triggering mechanisms activated at the late phase of the UPR. The MGST2-LTC_4_ pathway is regulated both by induction of its components and more importantly, by their translocation and co-localization at the nucleus. Such co-localization ensures effective production of LTC_4_ at its site of action. Binding of LTC_4_ to its internalized receptors then sets in motion nuclear and perinuclear translocation of NOX4, generating ROS, oxidative nuclear DNA damage, apoptosis and necrosis. This sequence of events is in line with the well-documented activation of NOX4 by oncogenic H-Ras, which leads to production of nuclear ROS, subsequent DNA damage and apoptosis^20^,^32^.

Compared with their structurally related prostaglandins, much less is known about non-immunological functions of leukotrienes. Exogenous LTC_4_ was shown to induce angiogenesis and endothelial cell proliferation (reviewed in ref. 47). In contrast, we show that stress-induced intracrine LTC_4_ triggers oxidative DNA damage and cell death. NOX4-generated ROS also elicit opposite cellular responses. High levels of NOX4-derived ROS trigger DNA damage and cell death, whereas low levels of ROS, and particularly hydrogen peroxide, serve as a cue for cell proliferation^48^,^49^. Similarly, extensive hypoxia, nutrient shortage and accumulation of toxic metabolites trigger ER stress and subsequent cell death, manifested as necrotic cores in rapidly growing solid tumours, whereas low levels of continuous ER stress were shown to send proliferative signals in such tumour cells^50^,^51^,^52^. Our finding that LTC_4_ receptor antagonists greatly reduced the level of NOX4 under extensive ER stress but had no effect on its basal expression level is in line with the dual opposite role of NOX4-generated ROS. The reduced level of NOX4 under ER stress in the presence of LTC_4_ receptor antagonists (Fig. 2j and Supplementary Fig. 3c) suggests that signalling by the LTC_4_ receptors is required for preventing ER stress-triggered degradation of NOX4 in these cells.

The MGST2-LTC_4_ pathway is downstream of CHOP and is further upregulated by a positive feedback loop between CHOP and MGST2. Induction of CHOP by MGST2 is further positively regulated by NOX4, since knockdown of NOX4 attenuates CHOP expression^53^. The almost complete inhibition of ER stress-triggered ROS generation by knockdown of *Mgst2* and by LTC_4_ receptor antagonists indicates that the CHOP-activated MGST2-LTC_4_ pathway is the major trigger of oxidative stress and DNA damage under ER stress. In addition, this pathway significantly contributes to ER stress-triggered cell death. Upregulation of CHOP expression by LTC_4_ suggests that it may promote cell death not only by generating ROS but also by increasing other CHOP-mediated pro-apoptotic activities, such as regulating Bcl2, BAK and BAX. ER stress-triggered apoptosis may further promote oxidative stress by release of mitochondrial ROS^54^,^55^. Inhibition of the pro-survival enzyme MGST1 by LTC_4_ (ref. 56) may further promote cell death.

Doxorubicin triggers oxidative DNA damage of both mitochondrial and nuclear DNA^57^. Immunostaining of the dsDNA breaks-associated histone γ-H2AX indicated that the MGST2-LTC_4_ pathway is a critical mediator of the doxorubicin-triggered nuclear DNA damage. By attenuating chemotherapy-triggered ROS production and DNA damage, LTC_4_ inhibitors may find use in reducing the risk of secondary malignancies^58^,^59^. In particular, our findings that LTC_4_ inhibitors did not compromise the efficacy of chemotherapeutic agents in cells of haematopoietic lineage suggest that such agents may reduce the toxic side effects of chemotherapy when used in haematopoietic malignancies. Since NOX4 and ER stress have been implicated in additional major human pathologies, including metabolic diseases and neurodegeneration^60^,^61^,^62^, inhibition of its activity by LTC_4_ receptor antagonists may be of even broader clinical significance. Indeed, a recent study demonstrated that pranlukast attenuated the progression of Alzheimer's disease in a mouse model^63^.

In conclusion, the present study highlights a previously unrecognized MGST2-LTC_4_ pathway, elicited by ER stress. This pathway is the major mediator of ER stress and chemotherapy-triggered oxidative stress and oxidative DNA damage, acting through NOX4 and participating in ER stress-triggered cell death.

## Methods

### Cells and reagents

The murine B16 melanoma cells B16-F10 (ATCC CRL-6475), human amniotic WISH epithelial cells (CCL 25), human embryonic kidney cells (HEK 293T), human CCRF-CEM T-cell leukaemia lymphoblasts (ATCC CCL-119) and human U266 myeloma cells (ATCC TIB-196) were obtained from ATCC. WISH cells might be contaminated with HeLa cells, but that contamination has no effect on the conclusion of the study, as HeLa cells are also epithelial-like cells. Human HaCaT pre-keratinocytes were provided by P. Boukamp. WT and *Chop*-deficient mouse hepatocytes were provided by B. Tirosh. Primary MEFs were isolated from WT and *Mgst2*-deficient (129/Sv) mice and studied at passages 2 or 3. All cell lines were tested and found to be free of mycoplasma contamination. B16 cells, HEK 293T cells, HaCaT cells, WT and *Chop*-deficient mouse hepatocytes and primary MEFs were grown in DMEM. WISH cells were grown in MEM. CCRF-CEM and U266 cells were grown in RPMI 1640 medium. All media were supplemented with 10% foetal bovine serum, 50 U ml^−1^ penicillin and 50 μg ml^−1^ streptomycin. Leukotrienes and leukotriene inhibitors were purchased from Cayman. The following antibodies were used for immunofluorescence staining (IF) and for immunoblotting (IB): Anti MGST2 (HPA010707, 1:50 for IF and 1:250 for IB), anti HMGB1 (H9537, 1:1,000 for IB) and anti FLAG M2 (F3165, 1:2,500 for IB) were from Sigma-Aldrich; anti LTC_4_ (ADI-905-902, 1:100 for IF) was from Enzo; anti 5-LO (#610695 and ab39347, 1:50 for IF and 1:250 for IB) was from BD Transduction Laboratories and Abcam, respectively; anti NOX4 (#3187-1, 1:50 for IF and 1:1,000 for IB) and anti NOX2 (gp91-phox #5653-1, 1:1,000 for IB) were from Epitomics; anti CysLTR1 (ab93481, 1:50 for IF and 1:1,000 for IB), anti FLAP (ab79923, 1:50 for IF), anti cPLA2 (ab58375), anti Lamin A (ab8980, 1:1,000 for IF), anti PDI (AB2792, 1:100 for IF) and anti γ-H2AX (ab2893, 1:1,000 for IF) were from Abcam; anti CHOP (GADD 153, sc-793, 1:500 for IB) (R-20) and anti CysLTR2 M-42 (sc-98863, 1:50 for IF and 1:200 for IB) were from Santa Cruz Biotechnology; anti 8-OHdG (12501, 1:5,000 for IF) was from QED Bioscience; anti cleaved caspase-3 Asp 175 (#9661, 1:500 for IB) was from Cell Signaling; anti-Actin (#69100, 1:10,000 for IB) was from MP Biomedicals. The secondary antibodies goat anti-rabbit IgG-H&L (Cy3; ab6939, 1:1,000 for IF) and rabbit anti-mouse IgG- H&L (HRP, ab6728, 1:10,000 for IB) were from Abcam. Alexa Fluor-488-labelled goat anti-mouse IgG (A-11001, 1:1,000 for IF) and goat anti-rabbit IgG (A-11008, 1:1,000 for IF) secondary antibodies were from ThermoFisher Scientific. All other reagents and media were from Sigma-Aldrich.

### Cell culture, induction of ER stress and inhibition of LTC_4_

Cell culture media were supplemented with 10% foetal bovine serum and antibiotics (DMEM-10 and MEM-10) and grown in humidified 8% CO_2_ incubator at 37 °C. ER stress and cellular cytotoxicity were elicited by treating cells for 24 h with Tm (0.75 μg ml^−1^), BfA (0.66 μg ml^−1^), Tg (50 nM), MG262 (0.2 μg ml^−1^), bortezomib (25 ng ml^−1^), doxorubicin (3 μM), 5-FU (0.125 μg ml^−1^) or vincristine (0.05 μM), unless otherwise stated. Leukotriene inhibitors were used at the following concentrations: MK571, 10 μM; reversan, 20 μM; zileuton, 12.5 μM; BAY u9773, 1 μM; pranlukast, 10 μM; BAY cysLT2, 10 μM; montelukast, 5 μM, unless otherwise stated.

### Immunoblotting

Cells were seeded in 6-well plates (800,000 cells per well) and grown for 24–48 h before treatments. Following treatments the cultures were washed three times with ice-cold phosphate-buffered saline (PBS) and the cells were collected with trypsin-EDTA. Cell pellets were re-suspended in two packed cell volumes of RIPA lysis buffer (50 mM Tris-HCL, pH 7.5, 150 mM NaCl, 5 mM EDTA, 1% Triton X-100, 0.5% Sodium deoxycholate, 0.1% SDS). The re-suspended pellets were kept on ice for 20 min. The clarified (14,000*g*, 20 min.) lysates were collected and stored at −80 °C. Clarified culture supernatants (40 μl) were used for IB of HMGB1 in the culture supernatants, and Ponceau s staining was used as control for equal loading. The bicinchoninic acid (BCA) Protein assay reagent kit (Pierce) was used for measuring protein concentration, using bovine serum albumin as a standard. Protein samples were boiled in SDS–polyacrylamide gel electrophoresis sample buffer containing 25 mM dithiothreitol, and the supernatants were resolved by gradient SDS–polyacrylamide gel electrophoresis (7.5–12% acrylamide). Proteins were then transferred onto a nitrocellulose membrane, which was incubated with the indicated primary antibodies. The Super Signal Detection Kit (Pierce) was used for visualizing the primary antibodies. Mouse MGST2 could not be detected by IB with any of the commercially available antibodies. Images of whole immunoblots are shown in Supplementary Fig. 10.

### IF staining

Cells were cultured (8 × 10^5^ per 2 ml or 3 × 10^5^ per 300 μl medium, 24 h) on cover slips for confocal microscopy (bar size=5 μm) or in μ-slide 8-wells (ibidi) for fluorescence microscopy (other bar sizes). The cultures were treated as described and then fixed using 4% paraformaldehyde, 0.5% Triton X100 in PBS for 2 min, followed by paraformaldehyde (4% in PBS, 20 min). The slides were washed with PBS (15 min), blocked with bovine serum albumin (2% in PBS, 30 min) and washed with PBS (5 min). The slides were then stained with primary antibody (in PBS, 45 min), washed with PBS (15 min) and incubated in the dark with Alexa 488-labelled second antibody (1:1,000, 45 min.), or with Cy3-labelled secondary antibodies (1:1,000, 45 min). For LTC_4_ IF staining, the cultures were treated as described in the presence of the MRP1 transporter reversan (10 μM). The cultures were then fixed with 1-ethyl-3-(3 dimethylaminopropyl) carbodiimide (EDAC, 1% in HBSS, 1 h), blocked with bovine serum albumin (2% in HBSS) and stained with rabbit anti LTC_4_ (1:100, 1 h), followed by Alexa-488-labelled second antibody (1:1,000, 1 h, ref. 64). Cell nuclei were stained with Hoechst 33258 (2.5 μg ml^−1^, 5 min).

### ROS staining

Staining of ROS was performed with the superoxide anion indicator, dihydroethidium (5 μM, 30 min). The cells were then washed three times with PBS, and nuclei were counterstained with Hoechst 33258 (5 μM, 5 min). Alternatively, cells were stained with dichloro-dihydro-fluorescein-diacetate (DCFH-DA, 10 μM, 40 min) and then washed three times with PBS. Nuclei were counterstained with Hoechst 33258 (5 μM, 5 min). Cells were then immediately observed under a fluorescence microscope and photographed.

### Immunohistochemistry

Mouse kidney sections were fixed in 4% paraformaldehyde, embedded in paraffin and sections of 5 μm were prepared. The sections were de-paraffinized and rehydrated in warm 10 mM sodium citrate, pH 6.0. Sections were then stained with haematoxylin-eosin, or immunostained with antibodies to Aminopeptidase A (ab36122, Abcam, 1:150), MGST2 (Atlas Antibodies HPA010707, 1:50), cleaved caspase-3 (Asp_175_, Cell Signaling #9661, 1:300), NOX 4 (Abcam ab109225, 1:100) or 8-OHdG (QED Bioscience 12501, 1:200). The sections were then incubated with biotin-conjugated secondary anti-rabbit IgG antibody (711-065-152, 1:200) or anti-mouse IgG (715-065-151, 1:100), (both from Jackson ImmunoResearch Laboratories), followed by incubation with avidin-biotin-peroxidase complex (ABC kit, Vectastain, Vector laboratories). Cell nuclei were counterstained with haematoxylin. The sections were then dehydrated and stabilized with Entellan mounting medium (Merck-Millipore).

### Image analysis

Staining intensities were measured using the ImageJ program (NIH) and normalized to nuclei counts. Fields containing at least 300 nuclei were analysed in triplicates. Damage to the kidney proximal tubules was determined by selecting the entire proximal tubular areas using the lasso tool of Photoshop, and vacuoles were counted using the ImageJ program. The extent of protein co-localization and nuclear localization was quantitated in confocal microscopy images using the Coloc 2 algorithm of FIJI-ImageJ (ref. 65). Per cent NOX4 or 8-OHdG immunostained nuclei was determined by the ratio of immunostained nuclei and Hoechst 33258-stained nuclei.

### RNAi

Cells in 6-well or in 96-well plates in media lacking antibiotics were transfected for 48 h with siRNA pools (siGENOME, Dharmacon RNAi Technologies) directed against murine *Mgst2* mRNA (NM_174995.2), human *Nox4* (NM_016931.1) or human *Chop* mRNA (NM_001195053.1), using DharmaFECT 1 reagent (Dharmacon) according to the manufacturer's protocol. ER stress inducers were added at 48 h post-transfection.

### Reverse transcription and real-time PCR

Total RNA (1 μg), isolated from cells using PerfectPure RNA Cultured Cell Kit (5 Prime) was reverse transcribed using random hexamers and Superscript II RNase H^−^ Reverse Transcriptase (Invitrogen). The reaction mixtures were diluted 20-fold, and 4.5 μl was then used as the template for regular PCR or real-time PCR. Human *Mgst2* mRNA (Nm_002413.3) specific primer pair combinations were: forward: 5′-AAAGATGGCCGGGAACTC-3′. Reverse: 5′-ATCTTGCCTTTCCAACTTGC-3′. Human *Chop* mRNA (*DDIT3*, NM_004083.4) specific primer pair combinations were: forward: 5′-AAGGCACTGAGCGTATCATGT-3′. Reverse: 5′-TGAAGATACACTTCCTTCTTGAACAC-3′. Human TATA box binding protein (*TBP*) mRNA served as a reference transcript, using the following specific primer pair combination: forward: 5′-CCCATGACTCCCATGACC-3′. Reverse: 5′-TTTACAACCAAGATTCACTGTGG-3′.

These probes were designed using Roche ProbeFinder Software Version 2.35 (Roche Diagnostics). Quantitative PCR was performed using a Roche LightCycler 480 real-time PCR System, and TaqMan Universal PCR Master Mix (Applied Biosystems). The reactions (10 μl final volume) contained 0.25 μM primers (Sigma-Aldrich) and 0.1 μM probe (No. 13 for murine *Mgst2* mRNA, No. 18 for human *Mgst2* mRNA, No. 21 for human *Chop* mRNA and No. 51 for *TBP* mRNA, Roche). The amplification programme was: initial denaturation at 95 °C for 15 min, followed by 45 cycles of 95 °C for 15 s, 60 °C for 1 min and 40 °C for 30 s. Gene expression level was normalized to the *TBP* reference mRNA. The fold change in gene expression compared with mRNA from control cells was calculated using LightCycler Software version 4.05. The results are presented as the mean±s.d. of triplicates.

### Cell viability assays

Adherent cell viability was determined either by Crystal violet staining or by neutral red staining in 96-well plates^66^. Cell cultures in 96-well plates were fixed and stained with 5% crystal violet in 66% aqueous methanol. Staining intensities were determined with an enzyme-linked immunosorbent assay (ELISA) reader at 570 nm. Alternatively, cell viability was measured by neutral red staining. Briefly, cells were incubated with neutral red (70 mg l^−1^ in 0.1 ml DMEM-10, 37 °C, 30–60 min), washed 3X with PBS, re-suspended in lysis buffer (0.1 ml) and OD was measured with an ELISA reader at 540 nm. Calibration curves were prepared by seeding known amounts of cells onto 96-well plates. After 6 h, the cells were stained with neutral red and the OD was read as described above. Viability of cells growing in suspension was determined using WST-1 (Roche) and was determined following 2 h incubation with an ELISA reader at 450 nm.

### Stable and inducible *Mgst2* overexpression

A cDNA encoding human *Mgst2* (h*Mgst2*) fused to a FLAG tag at the N-terminus (Epoc Life Science) was inserted into pcDNA4/TO (Life Technologies) to generate pcFLAG-h*Mgst2*. HEK 293T cells (3 × 10^5^ cells per 2 ml DMEM) were cultured for 24 h and then transfected with pcDNA4/TO or pcFLAG-h*Mgst2* (1 μg ml^−1^) using jetPEI (2 μg ml^−1^, Polyplus-transfection) as a transfection reagent. After 24 h, the cells were trypsin-digested and analysed by IB with anti-FLAG antibody, or seeded at 5 × 10^4^cells per 100 μl DMEM-10 on poly-D-lysine-coated 96-well plates for further experimentation. HEK 293T clones stably expressing Tet-inducible h*Mgst2* were generated by co-transfection with pcFLAG-h*Mgst2* and pcDNA6/TR (Life Technologies) and selection with zeocin (350 μg ml^−1^) and blasticidin (1 μg ml^−1^). Viable clones were isolated and tested for doxycycline-induced *hMgst2* over-expression by IB with anti-FLAG antibody.

### Generation and genotyping of *Mgst2*-deficient mice

*Mgst2*-targeted ES cells were obtained from the 129/SvEvBrd (129/Sv) mouse strain gene trap library of ES cells of the Texas Institute for Genomic Medicine (TIGM, College Station, TX). ES cells were aggregated with zona free morulae from outbred ICR mice, and the resulting blastocysts were transferred into the oviducts of pseudo-pregnant females for gestation^67^. Chimeric mice were identified by the presence of agouti coat colour. Germ-line transmission of male chimeras was verified by mating to ICR females. Chimeric males were mated with 129/Sv females to generate F1 offspring. Germ-line transmission of the target allele was confirmed by PCR as follows: tails were cut from all offspring and genomic DNA isolated by DirectPCR Lysis Reagent (Tail, Viagen Biotech), containing freshly prepared proteinase K (0. 125 mg ml^−1^, 17 h at 55 °C followed by 45 min at 85 °C, Sigma-Aldrich). The genomic DNA was amplified with two pairs of primers for the wild type and the mutant alleles:

Wild-type forward primer: 5′-GCTGTGGTCATGTGACAAGG-3′.

Wild-type reverse primer: 5′-TCCTTCCCCCTCTCTCTGTT-3′.

Mutant forward primer: 5′-CTTGCAAAATGGCGTTACTTAAGC-3′.

Mutant reverse primer: 5′-TGGGTGGGATAAGGGTTACA-3′.

The PCR conditions were: initial 4 min denaturation at 95°C, then 32 cycles of 95°C for 40 s, 58°C for 30 s and 72°C for 60 s. Then, the last extension was performed at 72°C for 5 min.

The *Mgst2*-deficient mice bred normally and appeared indistinguishable from their wild-type littermates, with no significant differences in food intake or body weight. Haematoxylin–eosin staining of liver, kidney and heart tissue sections also revealed no significant morphological differences.

### Animal studies

The Animal Care and Use Committee of the Weizmann Institute of Science approved all experimental procedures involving animals. The following studies were performed:

### Mouse model of Tm-induced acute kidney injury

Experiments were performed on WT and *Mgst2*-deficient 129/Sv female mice (10–12-week-old, weighing at least 20 g). The mice were housed with a 12:12-h light–dark cycle and with free access to standard diet and water. One group of WT mice and one group of *Mgst2*-deficient mice received a single intraperitoneal (ip) injection of Tm (1.5 mg kg^−1^ body weight) in 150 mM glucose. Control WT and *Mgst2*-deficient mice received vehicle alone. The animals were observed twice daily and euthanized painlessly with CO_2_ immediately upon showing either 20% reduction of body weight or two other obvious signs of distress, including lethargy, ruffled fur, shaking, paralysis or confused movements. Four days after Tm administration the mice were euthanized, kidneys were removed, immersed in 10% neutral-buffered formaldehyde for 48–72 h. The tissues were paraffin embedded and processed for light microscopy. Sections were cut to a thickness of 5 μm and stained with haematoxylin–eosin or subjected to immunohistochemistry. To assess the extent of kidney injury, areas corresponding to the kidney cortex were selected from whole-kidney images and vacuoles ranging in size from 100 to 500 pixels and circularity ≥0.3 were counted using the ImageJ program. The per cent area of vacuoles versus the overall selected area is plotted. No vacuoles were observed in untreated WT or *Mgst2*-deficient mouse kidneys.

### Mouse models of Tm and chemotherapy-induced morbidity

Experiments were performed on WT and *Mgst2*-deficient 129/Sv mice as above. Female mice were given a single ip injection of Tm (2.5 mg kg^−1^ body weight, in 150 mM glucose). The animals were observed for signs of distress and euthanized as above. In another set of experiments, WT female mice (10 per group) were given Tm (1.5 mg kg^−1^, ip) once at time=0. Vehicle (1% DMSO in PBS) or pranlukast (1 mg kg^−1^ in 1% DMSO in PBS) were administered ip at time=0 and daily for 2 additional days. Mice were observed and euthanized as described above. Similarly, WT and *Mgst2*-deficient 129/Sv female mice (10 per group) were given a single ip injection of 5-FU (300 mg kg^−1^) at time=0 and observed for distress as above. In another set of experiments WT mice (9 female and 9 male per group) were given a single ip injection of 5-FU (300 mg kg^−1^) at time=0 and vehicle (1% DMSO in PBS) or pranlukast (1 mg kg^−1^ in 1% DMSO in PBS) were administered ip at days 0, 1, 2, 5, 6 and 7. The mice were observed for distress and kidneys were collected post mortem for histology and immunohistochemistry as above.

### Statistical analysis

Statistical analysis was performed using KaleidaGraph program (Synergy Software). All data were analysed using one-way analysis of variance (ANOVA) and *post hoc* Tukey's honest significant difference (HSD) test on at least three replicates. Data are shown as mean±s.d. for the indicated replicates. Data on survival of mice was analysed by the Gehan–Breslow–Wilcoxon test.

## Additional information

**How to cite this article:** Dvash, E. *et al.* Leukotriene C_4_ is the major trigger of stress-induced oxidative DNA damage. *Nat. Commun.* 6:10112 doi: 10.1038/ncomms10112 (2015).

## Supplementary Material

Supplementary Information Supplementary Figures 1-10
