# Supplementary material for: Leukotriene C4 is the major trigger of stress-induced oxidative DNA damage
Source: Nat Commun. 2015 Dec 11;6:10112. doi: 10.1038/ncomms10112 (PMC4682057; doi:10.1038/ncomms10112)
Supplement: Supplementary Information — Supplementary Figures 1-10 [file ncomms10112-s1.pdf]

Leukotriene C_4 is the major trigger of stress-induced oxidative DNA damage

Efrat Dvash, Michal Har-Tal, Sara Barak, Ofir Meir†, and Menachem Rubinstein

Supplementary Information

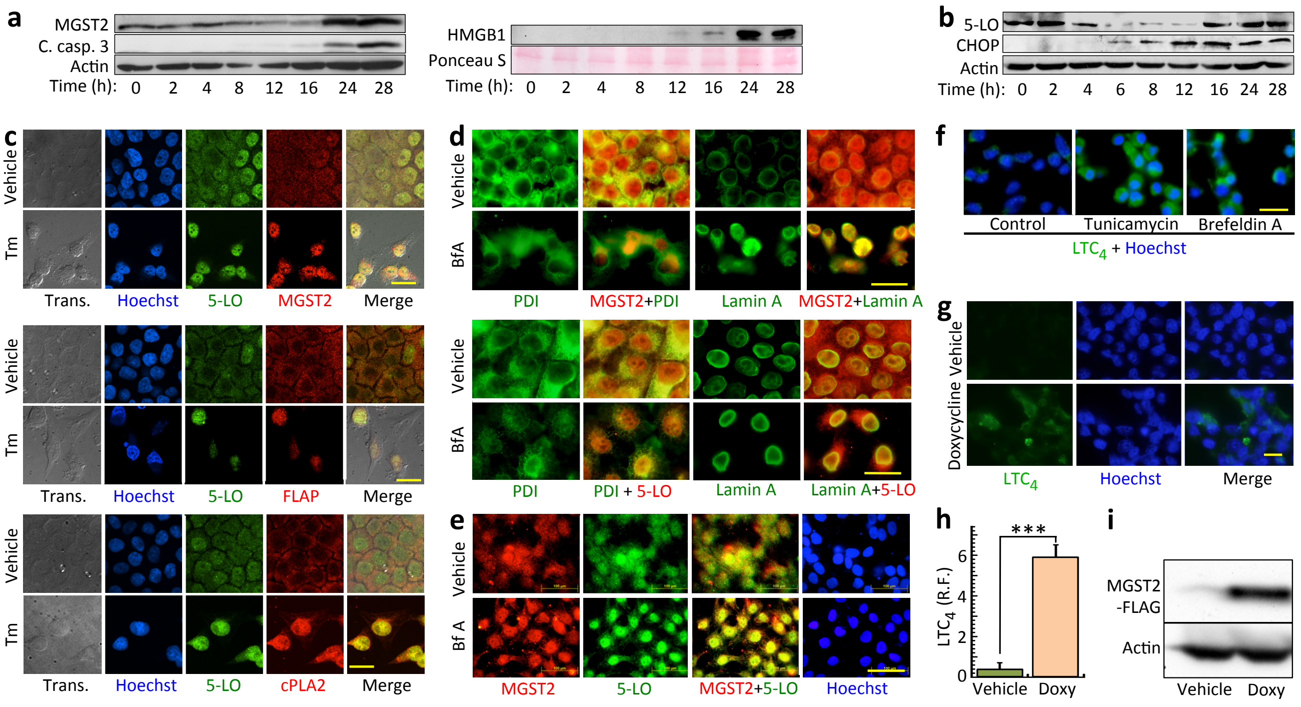

Supplementary Figure 1 | ER stress triggers expression, nuclear translocation and co-localization of the LTC_4 biosynthetic machinery. (**a**) Immunoblot of MGST2, cleaved caspase 3, and in extracts of human HaCaT pre-keratinocytes treated with BfA for the indicated times. HMGB1 released to the culture media was immunoblotted and Ponceau S staining served as loading control. (**b**) Immunoblot of the indicated proteins in extracts of mouse B16 cells treated with tunicamycin for the indicated times. (**c**) Imunostain of the indicated proteins in WISH cells treated with tunicamycin (Tm, 8 µg/ml, 48 h). The cultures were counter stained with Hoechst 33258 (Hoechst). Hoechst was excluded from the merged images. Trans. is light transmission. Bar=20 µm. (**d**) Immunostain of MGST2, 5-LO, the ER marker PDI and the nuclear lamina marker lamin A in HaCaT cells treated with BfA. Bar = 20 µm. (**e**) Immunostain of MGST2 and 5-LO in HaCaT cells treated with BfA. Bar = 100 µm. (**f**) Immunostain of LTC_4 in mouse B16 cells treated with tunicamycin (2 µg/ml) or BfA. Nuclei were counterstained with Hoechst. Bar = 20 µm. (**g, h**) Immunostain of LTC_4 in HEK 293T cells stably expressing Tet-inducible FLAG-tagged human MGST2, treated with doxycycline (Doxy, 2 µg/ml, 48 h). Bar = 20 µm. n=3, ***P=0.0002. Values represent means ± s.d. Statistical significance was determined using one-way ANOVA. R.F. is relative fluorescence intensity. (**i**) Immunoblot with anti-FLAG of MGST2-FLAG in extracts of 293T cells stably expressing Tet-inducible FLAG-tagged human MGST2, treated with doxycycline as in **g.** All images are representatives of three replicates.

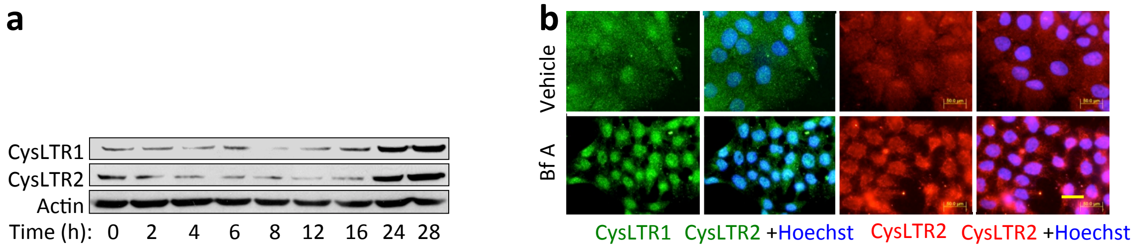

Supplementary Figure 2 | ER stress triggers expression, nuclear translocation and co-localization of the LTC_4 receptors. (**a**) Immunoblot of the indicated proteins in extracts of mouse B16 cells treated with tunicamycin for the indicated times. (**b**) Immunostain of CysLTR1and CysLTR2 in human HaCaT pre-keratinocytes treated with vehicle or BfA. Bar = 50 µm. Images are representatives of three replicates.

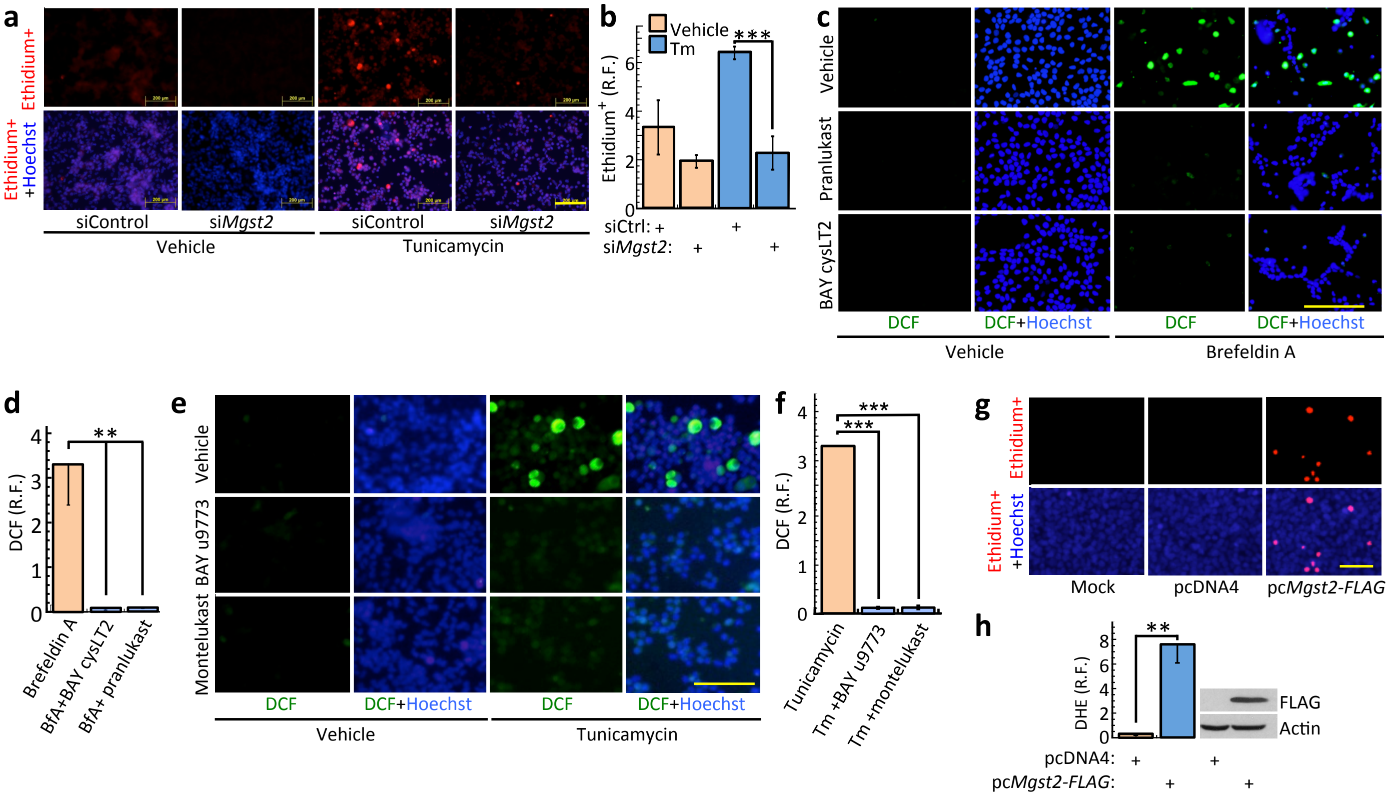

Supplementary Figure 3 | ER stress-generated LTC_4 triggers ROS accumulation. (**a, b**) ROS detection using dihydroethidium in mouse B16 cells transfected with control siRNA or *Mgst2* siRNA, and treated with tunicamycin (Tm). Bar = 200 µm. *n* = 3, ****P*<0.001. (**c, d**) ROS detection using DCFH-DA in HaCaT pre-keratinocytes treated with BfA in the absence or presence of pranlukast or BAY cysLT2. Bar = 500 µm. *n* = 3, ***P*<0.01. (**e, f**) ROS detection in mouse B16 cells treated with tunicamycin (1 µg/ml), in the absence or presence of BAY u9773 or the CysLTR1 antagonist montelukast. Bar = 200 µm. *n* = 3, ***P*<0.001. (**g**) ROS detection with dihydroethidium in HEK 293T cells 24 h after mock transfection, transfection with pcDNA4 or with pc*Mgst2*-FLAG. Bar = 500 µm. (**h**) Quantitation of ROS accumulation shown in (**g**), *n* = 4, ***P*<0.01. Shown also is an immunoblot with anti FLAG of HEK 293T cell extracts following transfection with control vector (pcDNA4) or with pc*Mgst2*-FLAG. This blot is a representative of three replicates. Values in **b, d, & f** represent means ± s.d. Statistical significance was determined using one-way ANOVA.

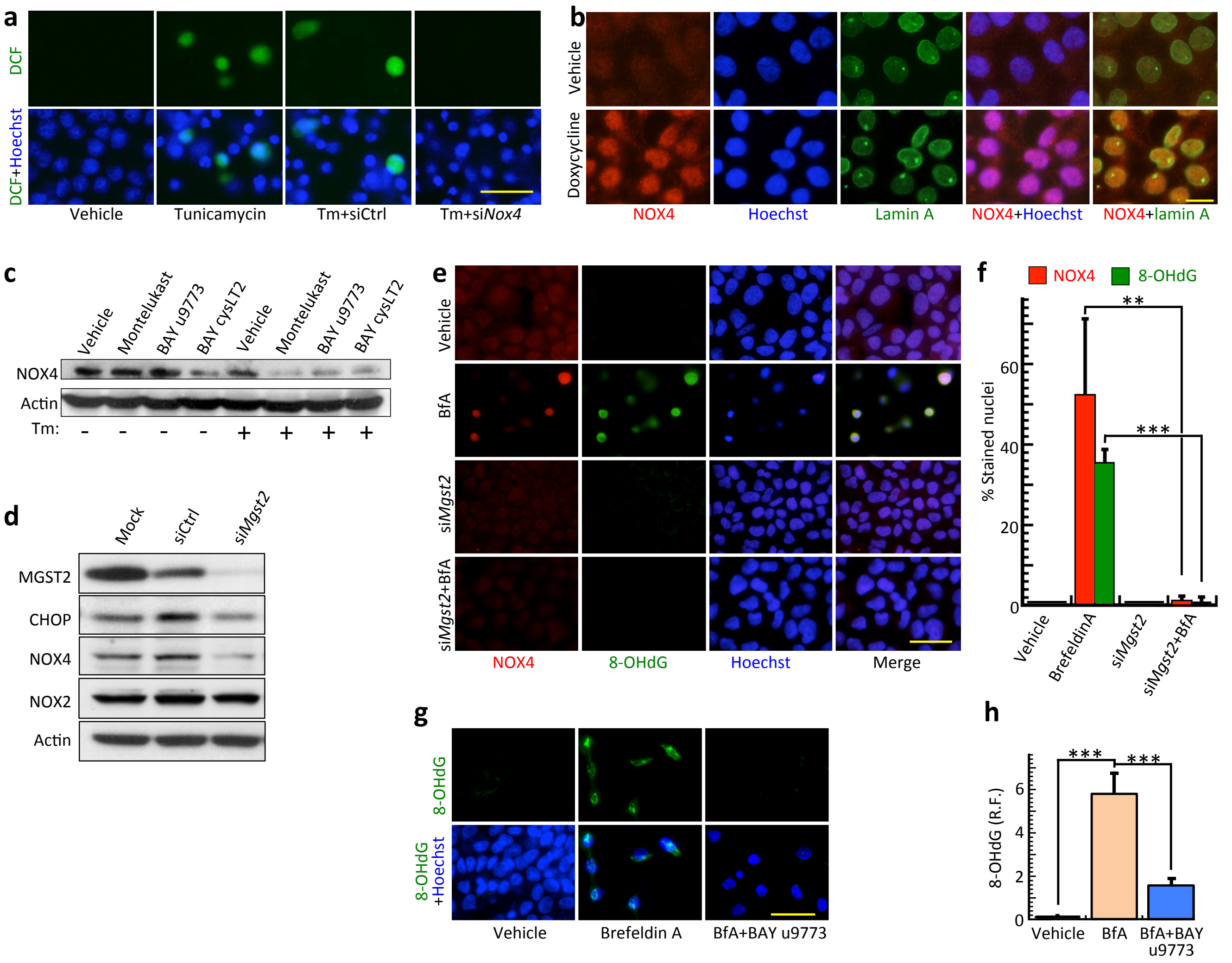

Supplementary Figure 4 | The MGST2-LTC_4 pathway mediates ER Stress-triggered nuclear translocation of NOX4 and oxidative DNA damage. (**a**) ROS detection using DCFH-DA in WISH cells transfected with control or *Nox4* siRNA and then treated with vehicle or tunicamycin (2 µg/ml, 48h). Bar = 50 µm. (**b**) Immunostain of HEK 293T cells stably expressing Tet-inducible FLAG-tagged human MGST2, treated with vehicle or doxycycline (2 µg/ml, 48 h), and immunostained with mouse anti lamin A and rabbit anti NOX4. Nuclei were counterstained with Hoechst 33258. Bar = 20 µm. (**c**) Immunoblot of NOX4 in extracts of mouse B16 cells treated with vehicle (-) or tunicamycin (Tm, 0.5 µg/ml, 24 h, +) in the absence (vehicle) or presence of the indicated LTC_4 receptor antagonists. (**d**) Immunoblot of the indicated proteins in extracts of WISH cells transfected with siCtrl or si*Mgst2* (48 h), followed by brefeldin A. (**e**) Immunostain of NOX4 and the oxidative DNA damage marker 8-OHdG in WISH cells transfected with siCtrl or si*Mgst2* (48 h), followed by vehicle or brefeldin A. All three images are merged. Bar = 50 µm. (**f**) Quantitation of percent nuclei stained with NOX4 and 8-OHdG in the cultures shown in **e**, $n = 4$, $**P<0.002$, $***P<0.001$. (**g, h**) Immunostain of 8-OHdG in HaCaT pre-keratinocytes treated for 48 h with brefeldin A in the absence (Vehicle) or presence of BAY u9773. Bar = 50 µm. $n = 3$, $***P<0.001$. Images **a, b, e** & **g** are representatives of three replicates. Values in **f** & **h** represent means ± s.d. Statistical significance was determined using one-way ANOVA.

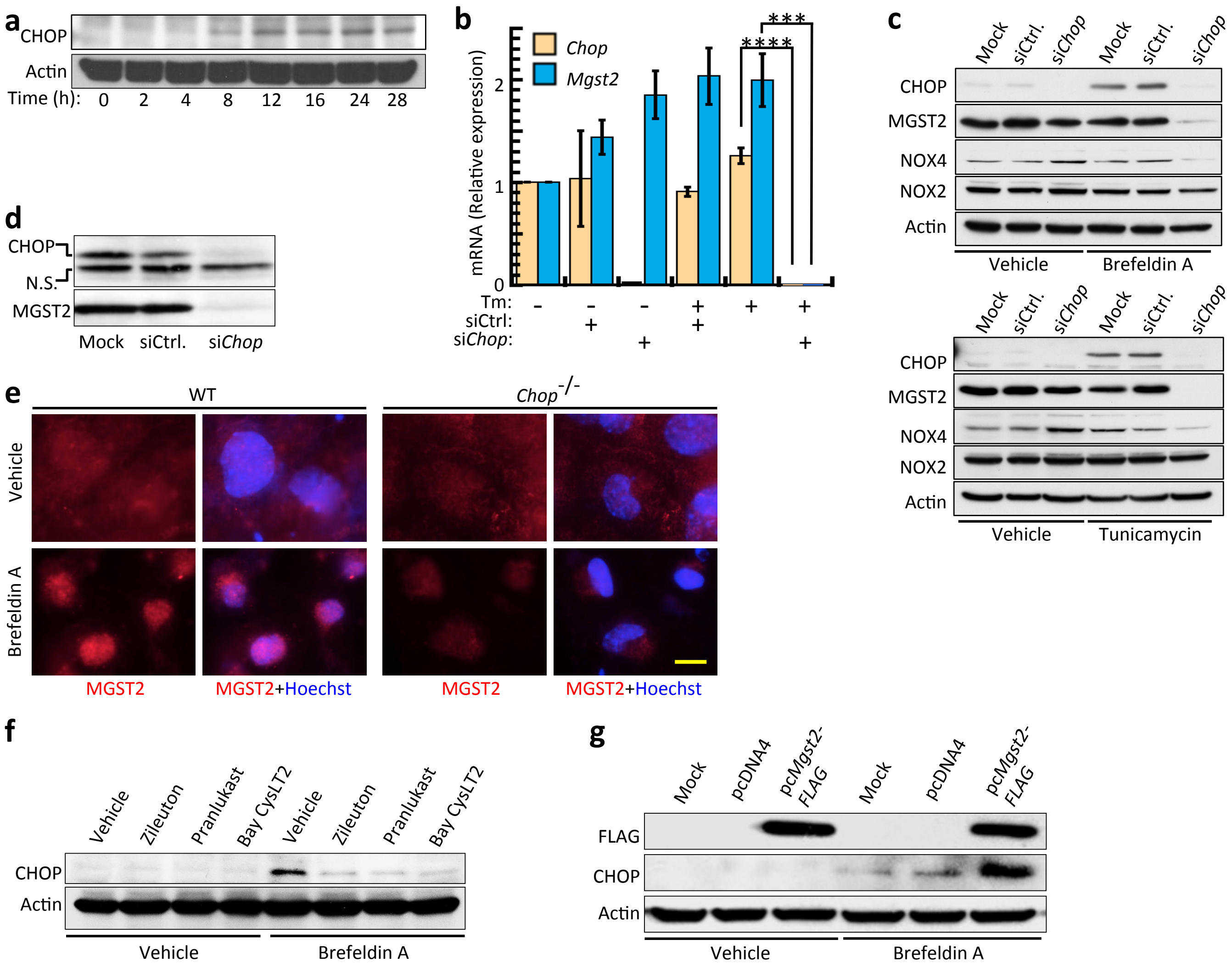

Supplementary Figure 5 | ER stress triggered-expression of MGST2 is downstream of CHOP. (**a**) Immunoblot of CHOP expressed in WISH cells at different times after induction of ER stress with brefeldin A. (**b**) qRT-PCR of *Chop* and *Mgst2* mRNA in extract of WISH cells mock transfected, transfected with control siRNA or *Chop* siRNA, followed by vehicle or tunicamycin (2 µg/ml, 48 h). *n* =4, ****$P= 9.3\text{x}10^{-6}$, *** $P= 1.82\text{x}10^{-5}$. Values represent means ± s.d. Statistical significance was determined using one-way ANOVA. (**c**) Immunoblot of the indicated proteins in extracts of human WISH cells following mock-transfection, transfection with control siRNA (siCtrl.) or with *Chop*-specific siRNA (si*Chop*) and induction of ER stress with brefeldin A (48 h) or tunicamycin as in **b**. (**d**) Immunoblot of MGST2 and CHOP in extracts of human HaCaT pre-keratinocytes following mock-transfection, transfection with siCtrl or si*Chop* and induction of ER stress with brefeldin A (48 h, N.S. is a non-specific band, serving as a load control. (**e**) Immunostain of MGST2 in immortalized WT and *Chop*-deficient mouse hepatocytes following induction of ER stress with brefeldin A (1 µg/ml 24 h). Bar = 10 µm. (**f**) Immunoblot of CHOP in extracts of WISH cell treated with vehicle or brefeldin A in the presence of vehicle, the 5-LO inhibitor zileuton, or the indicated LTC_4 receptor antagonist. (**g**) Immunoblot of CHOP and FLAG-tagged MGST2 in extracts of HEK 293T cells that were mock-transfected, transfected with empty vector (pcDNA4) or with pcFLAG-*Mgst2*, and then treated with vehicle or brefeldin A. Images and blots are representative of 3-5 replicates.

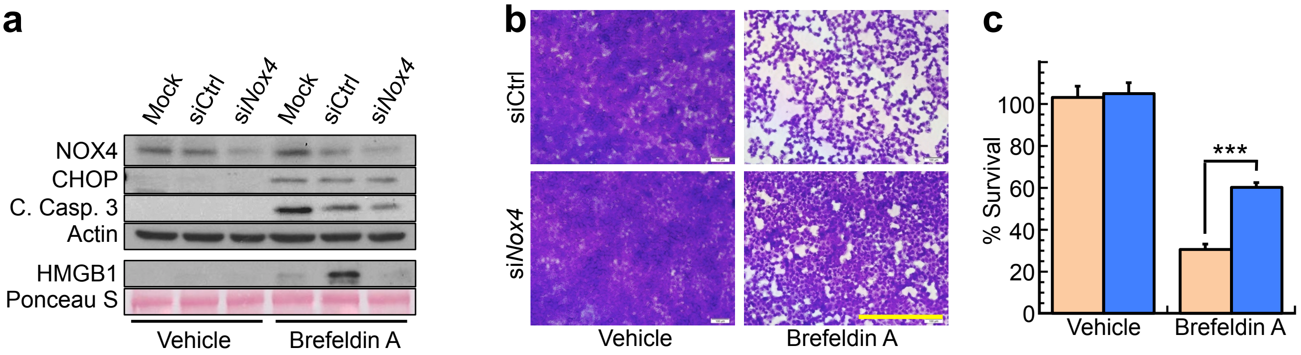

Supplementary Figure 6 | NOX4-mediated oxidative stress triggers cell death. (**a**) Immunoblot of the indicated proteins in extracts of WISH cells (top 4 panels) or HMGB1 in culture supernatants. The cells were transfected with the indicated siRNA followed by vehicle or brefeldin A. (**b**) Crystal violet stained cultures of WISH cells treated as in **a**. (**c**) Quantitation of cell viability in cultures of WISH cells shown in **b**. $n = 4$, ***P<0.0001. Values represent means ± s.d. Statistical significance was determined using one-way ANOVA.

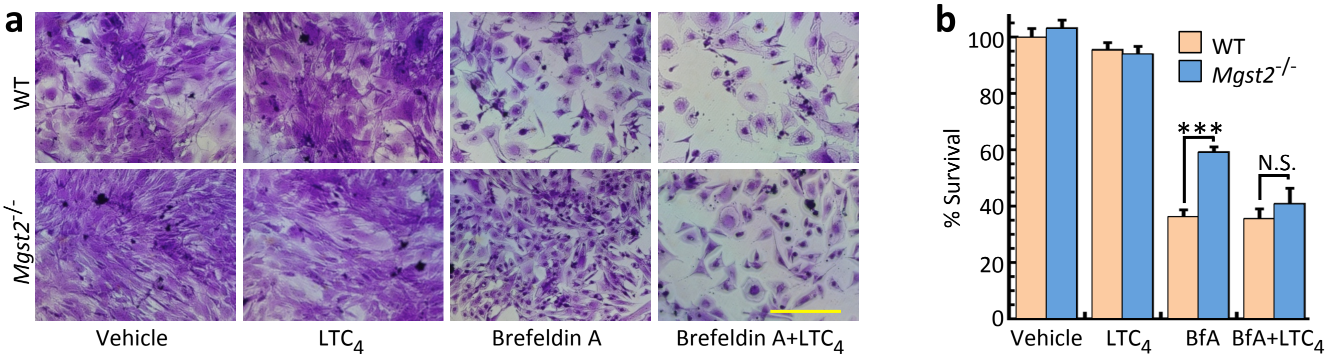

Supplementary Figure 7 | Exogenous LTC_4 restores the response of *Mgst2*-deficient mouse fibroblasts to ER stress and to chemotherapy. (**a, b**) Survival of WT and *Mgst2*-deficient MEFs at passage 2 following treatment with vehicle, LTC_4 (1 μM), brefeldin A, or brefeldin A and LTC_4 for 24 h. Bar=200 μm. $n = 3$, ***P<0.0001, N.S. not significant. Values represent means ± s.d. Statistical significance was determined using one-way ANOVA.

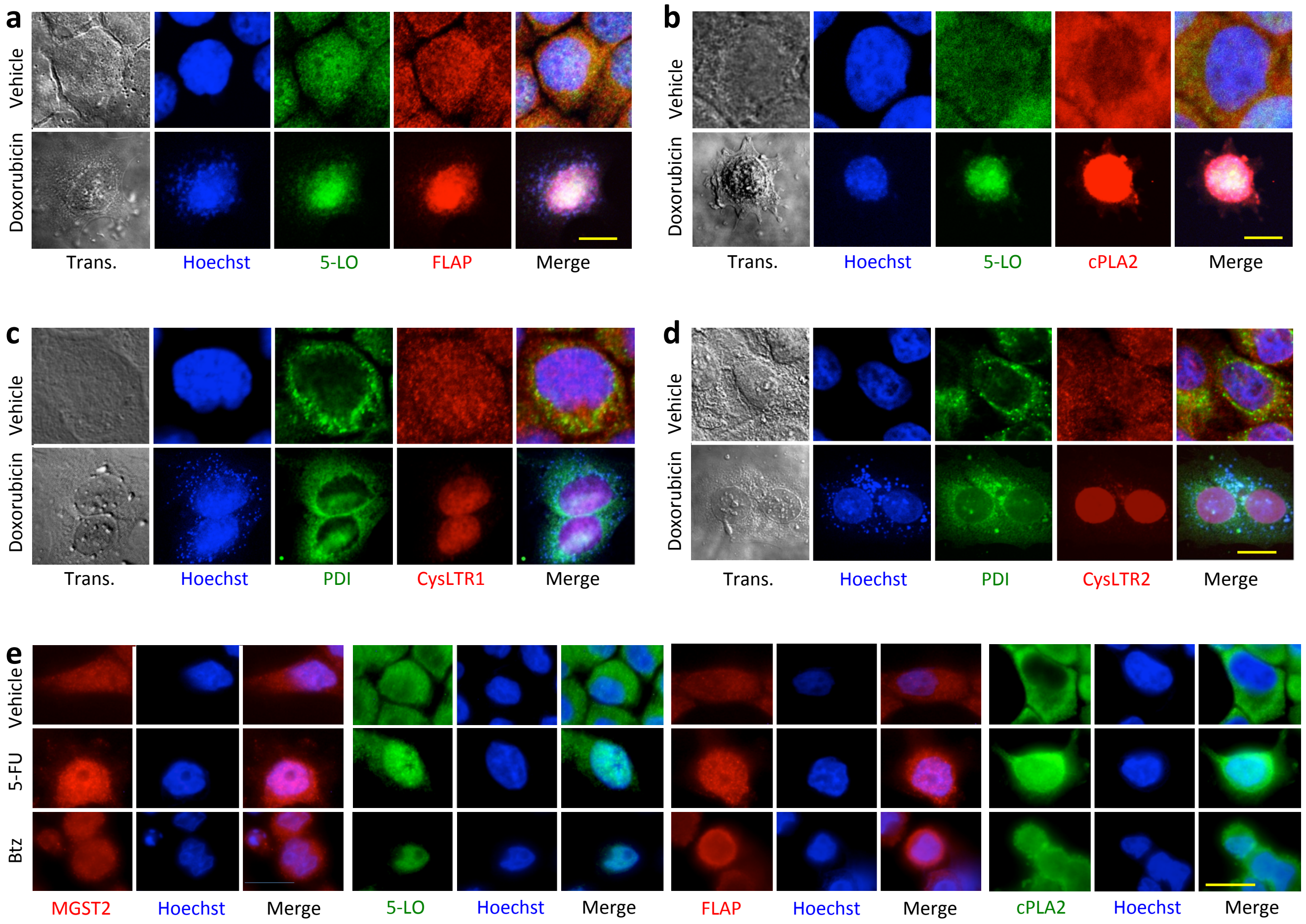

Supplementary Figure 8 | Chemotherapy triggers nuclear translocation of MGST2-based biosynthetic machinery of LTC_4. (a-d) Immunostain of 5-LO, FLAP, cPLA2, CysLTR1, CysLTR2 and the ER marker PDI in WISH cells treated with vehicle or doxorubicin (5 µM). Nuclei were counterstained with Hoechst 33258. Trans. is transmission light microscopy. All image channels except the transmission light microscopy (Trans.) were merged. Bars = 5 µm. (**e**) Immunostain of MGST2, 5-LO, FLAP and cPLA2 in WISH cells treated with vehicle, 5-FU or bortezomib (Btz) for 28 h. Bar = 10 µm. All images are representatives of three replicates.

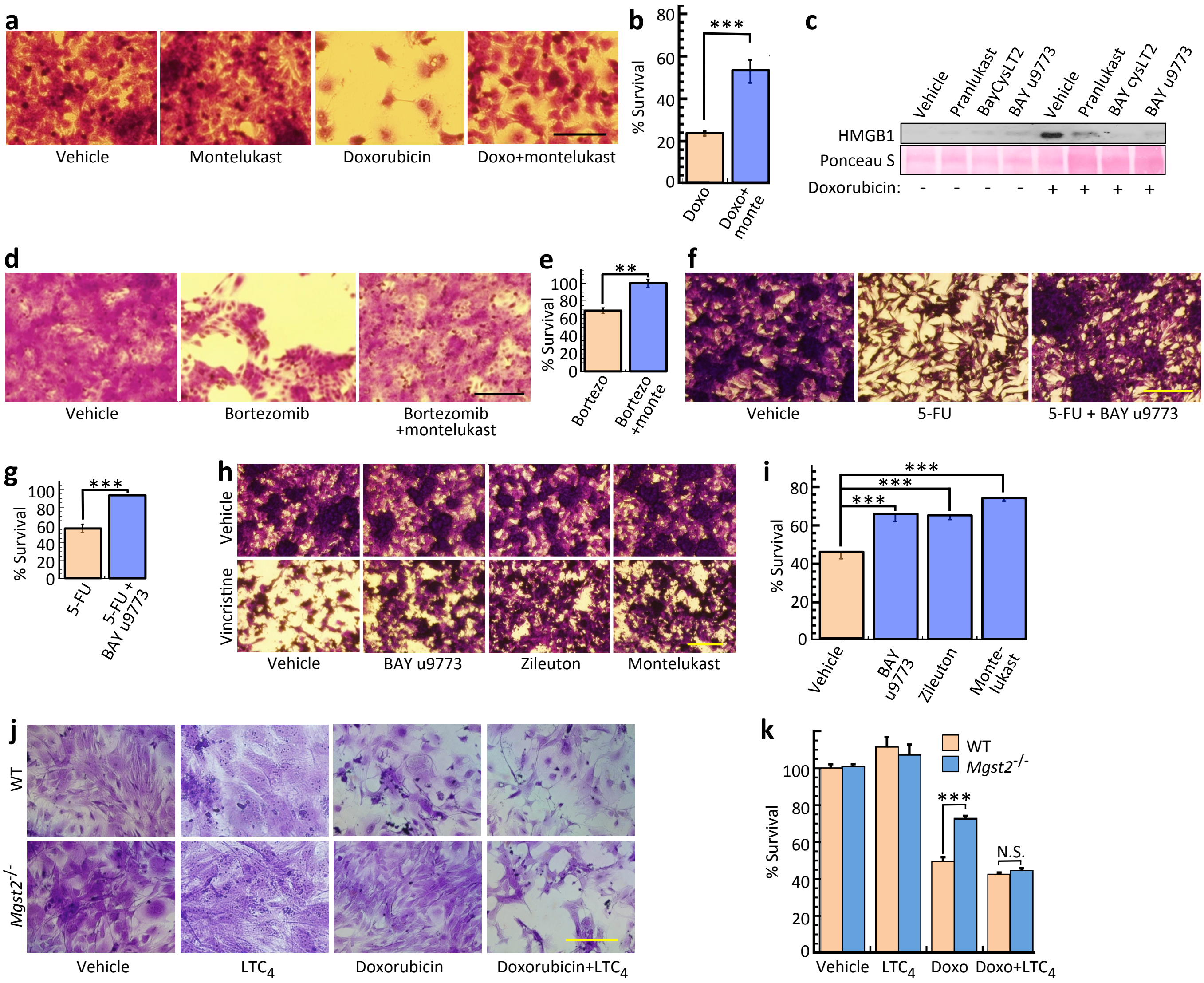

Supplementary Figure 9 | The MGST2-LTC$_4$ pathway mediates chemotherapy-triggered cell death. Percent survival was determined in all cultures shown in this figure following staining with crystal violet. (**a, b**) Survival of WISH cells treated with vehicle or doxorubicin in the absence or presence of montelukast (2.5 μM) for 72 h. Bar = 200 μm. $n = 4$, ***$P<0.001$. (**c**) Immunoblot of HMGB1 secreted to the culture supernatant of WISH cells treated with vehicle (-) or doxorubicin (5 μM, 28 h, +) in the absence or presence of the indicated LTC$_4$ receptor antagonists. This blot is a representative of three replicates. (**d, e**) Survival of HaCaT pre-keratinocytes treated with vehicle, bortezomib (bortezo) or bortezomib and montelukast (Monte). Bar = 500 μm. $n = 3$, **$P<0.02$. (**f, g**) Survival of B16 cells treated with vehicle, 5-FU, or 5-FU and BAY u9773 for 24 h. Bar = 500 μm. $n = 3$, $P<0.0001$. (**h, i**) Survival of B16 cells treated with vehicle or vincristine in the presence of vehicle, BAY u9773 (200 nM), zileuton or montelukast. Bar = 500 μm. $n = 4$, ***$P<0.0001$. (**j, k**) Survival of WT and *Mgst2*-deficient MEFs at passage 2 following treatment with vehicle, LTC$_4$ (1 μM), doxorubicin (5 μM), or doxorubicin and LTC$_4$ for 24 h. Bar=200 μm. $n = 3$, ***$P<0.0001$, N.S. not significant. Values in **b, e, g, i** & **k** represent means ± s.d. Statistical significance was determined using one-way ANOVA.

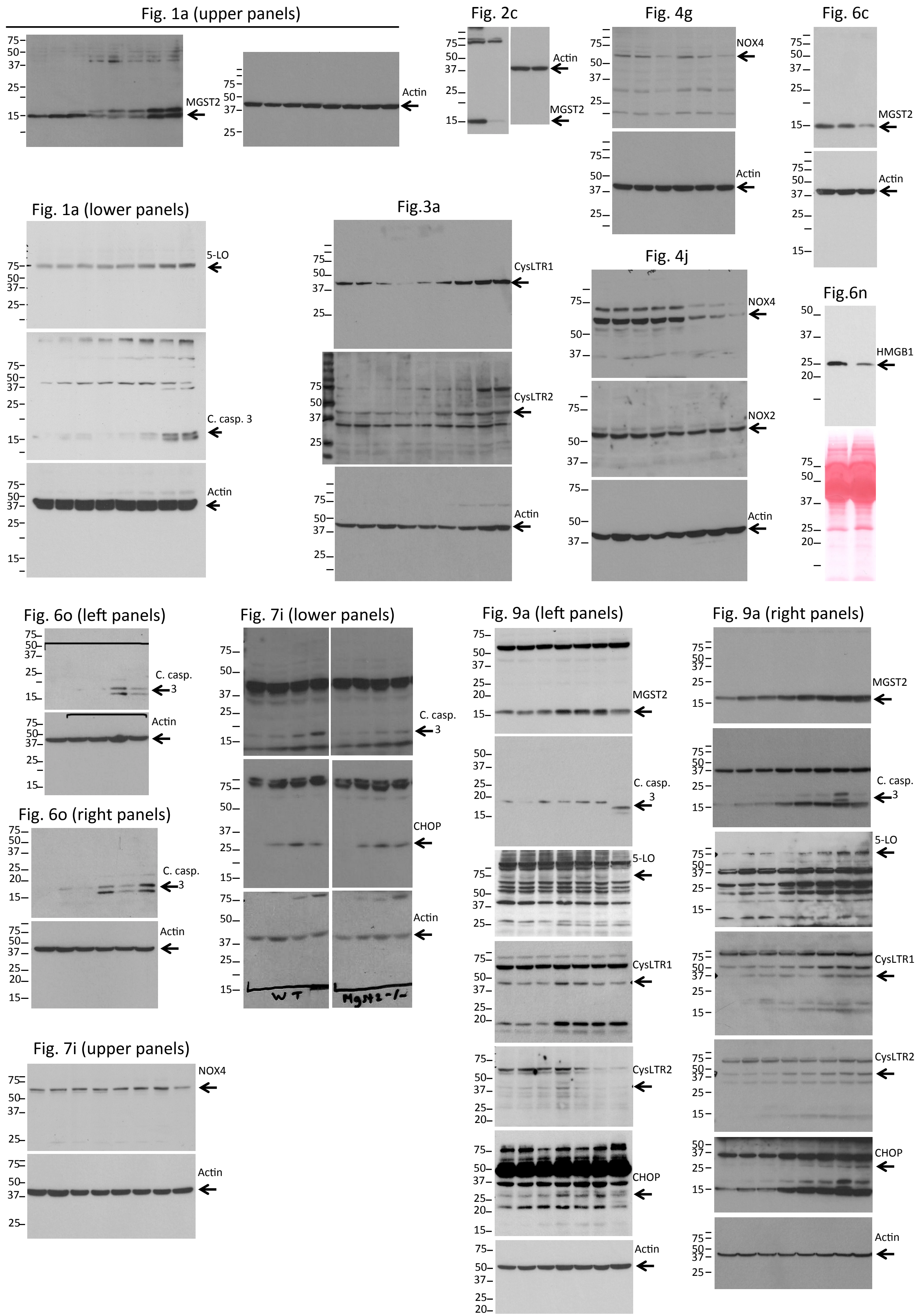

Supplementary Figure 10 | Immunoblots with molecular mass markers (in kDa).
